# Supplementary material for: Oxygen-independent organic photosensitizer with ultralow-power NIR photoexcitation for tumor-specific photodynamic therapy
Source: Nat Commun. 2024 Mar 21;15:2530. doi: 10.1038/s41467-024-46768-w (PMC10957938; doi:10.1038/s41467-024-46768-w)
Supplement: Supplementary file 1 — Supplementary Information [file 41467_2024_46768_MOESM1_ESM.pdf]

Supplementary information

Oxygen-independent organic photosensitizer with ultralow-power NIR photoexcitation for tumor-specific photodynamic therapy

Yufu Tang[1], Yuanyuan Li[2], Bowen Li[1], Wentao Song[1], Guobin Qi[1], Jianwu Tian[1], Wei Huang[2], Quli Fan[2], Bin Liu[1]**

[1]Department of Chemical and Biomolecular Engineering, National University of Singapore, Singapore 117585, Singapore. E-mail: cheliub@nus.edu.sg

[2]Key Laboratory for Organic Electronics and Information Displays and Jiangsu Key Laboratory for Biosensors, Jiangsu National Synergetic Innovation Center for Advanced Materials, Institute of Advanced Materials, Nanjing University of Posts and Telecommunications, Nanjing, 210023 China. E-mail: iamqlfan@njupt.edu.cn

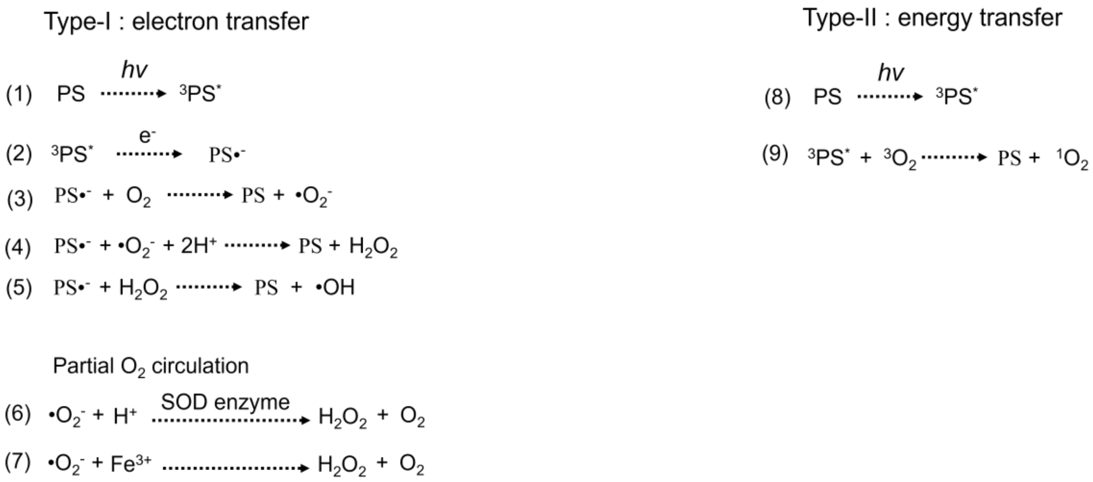

Supplementary Fig. 1 ROS generation mechanism: Type I electron transfer to form free radical ROS and hydrogen peroxide, and Type II energy transfer to form singlet oxygen.[1-7]

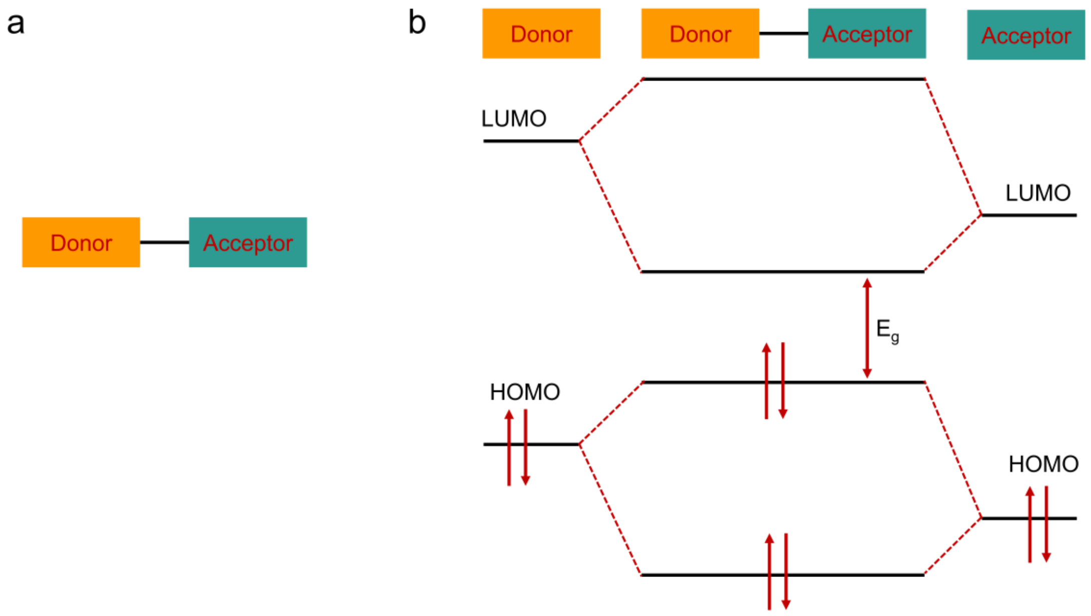

Supplementary Fig. 2 (a) Schematic of an organic donor–acceptor semiconductor, (b) hybridisation of the molecular orbitals of the donor and acceptors monomers of a generic organic semiconductor.

The energy levels of molecules can be tuned at the molecular level by chemical design. Most common types of conjugated polymers consist of an alternating electron-rich "donor" and an electron-deficient "acceptor" component, namely donor–acceptor (D–A) polymers (Fig. 2(a)). Perturbation theory dictates that the orbitals of donor–acceptor polymers hybridise by combining the HOMOs and the LUMOs of the constituent monomers, redistributing their energy levels and in turn the energy of the occupying electrons (Fig. 2(b)). This enables synthetic control and optimisation of these energy levels with relative ease and specificity. More specifically, by modifying the acceptor component, the LUMO energy level can be tuned to suit the application.

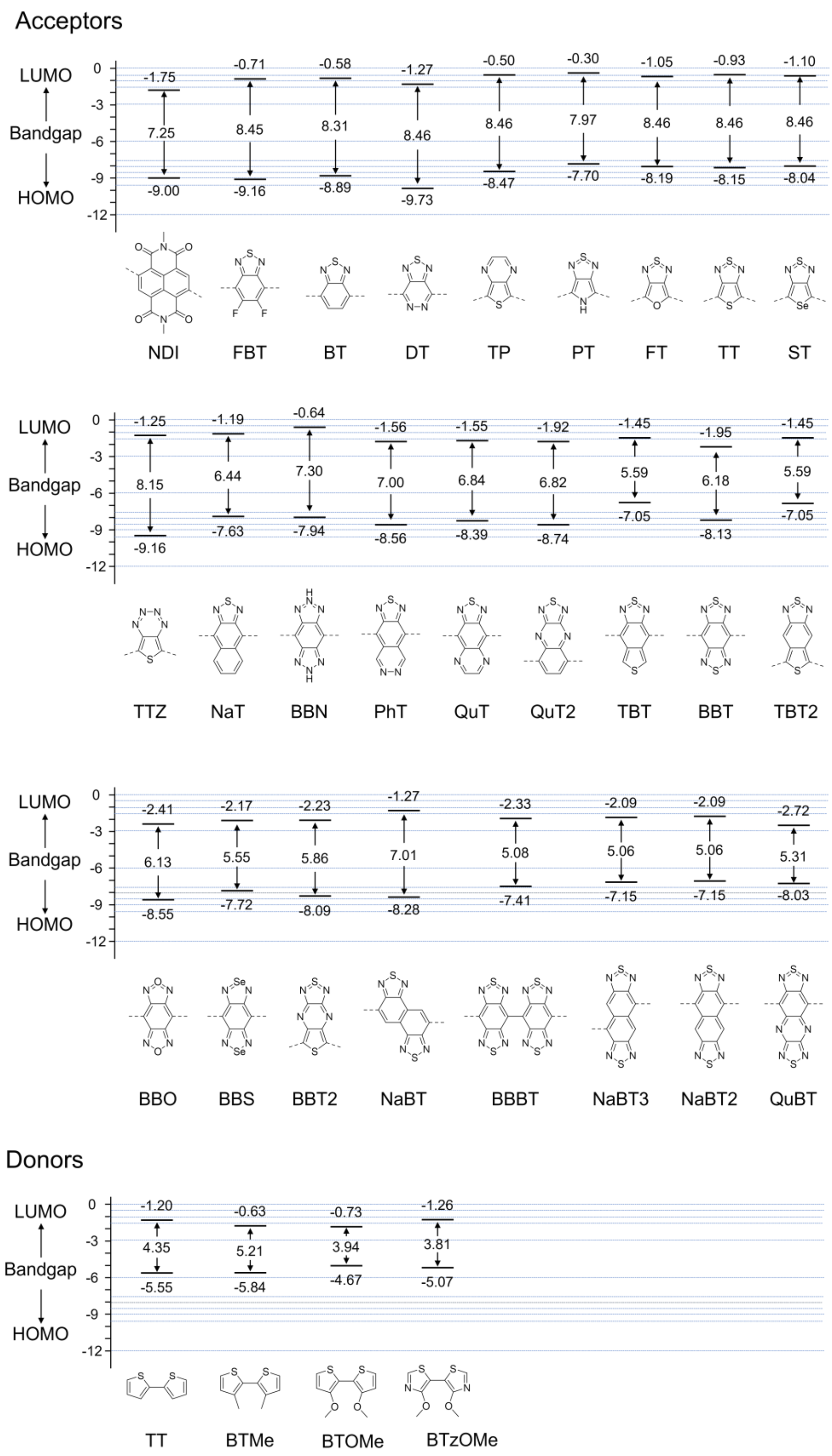

Supplementary Fig. 3 The HOMO and LUMO energy levels and corresponding HOMO-LUMO energy gaps (E_{gap}) of pure acceptors and donors.[8-10] Acceptors were calculated at the ωB97XD*/6-31G(d) level. Calculations of donors were carried out at the DFT//B3LYP/6-31G** level.

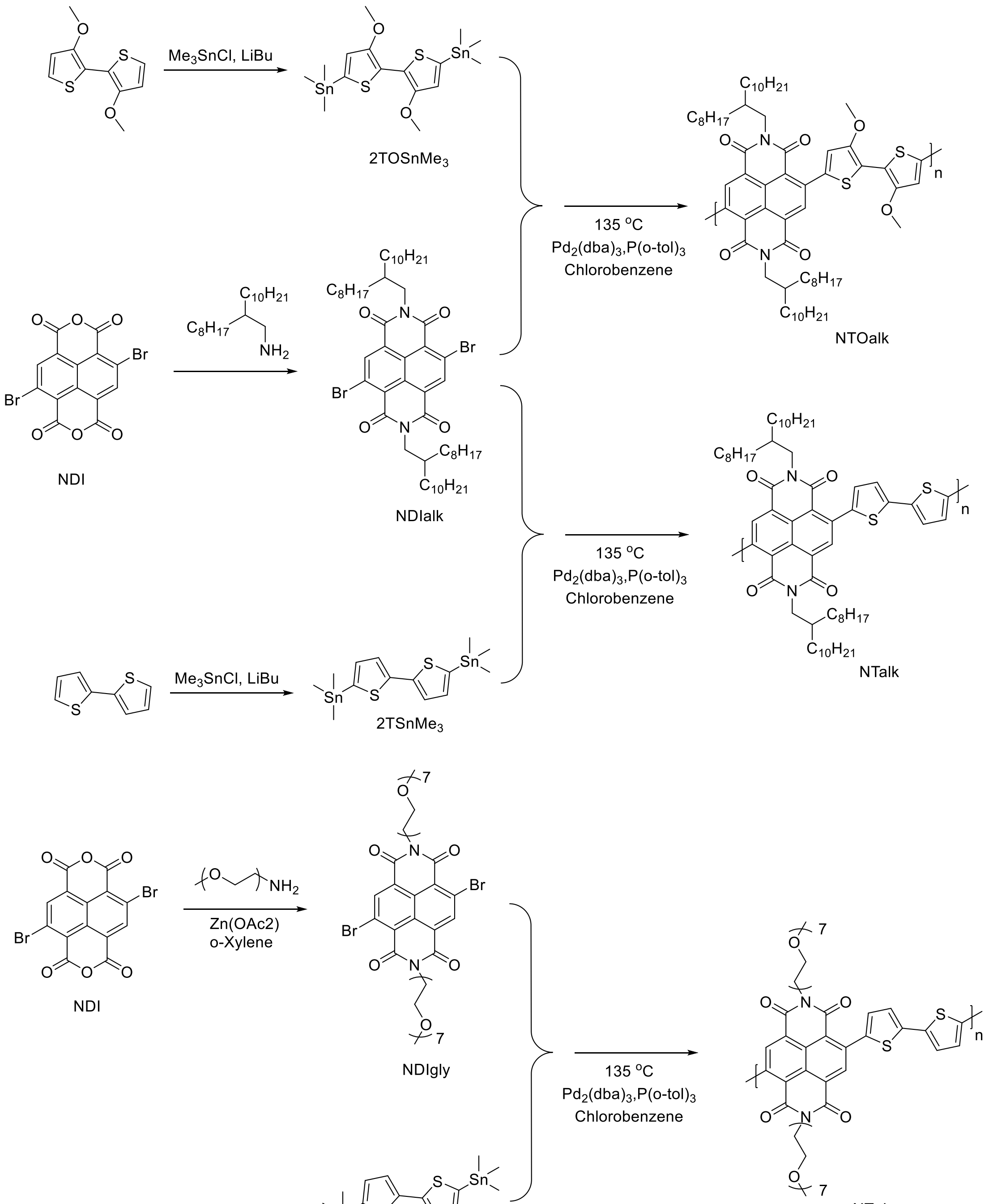

Supplementary Fig. 4 Overview of the synthesis of NTOalk, NTalk and NTgly.

Synthesis of 2TSnMe3. A solution of 2,2′-bithiophene (513 mg, 3.09 mmol, 1.00 eq.) in 100 mL of anhydrous tetrahydrofuran was cooled to -78 °C. 2.1 equiv of n-butyllithium (2.5 M in THF) was added slowly to a solution of 2,2'-bithiophene solution. The reaction mixture was stirred for 1 hour under an argon atmosphere. The solution was warmed to 0 °C. The trimethyltin chloride (2.2 equiv) was added to the solution. The mixture was stirred overnight at room temperature. The reaction mixture was diluted with ethyl acetate and the organic layer was washed with brine. The organic layer was dried over $MgSO_4$ and concentrated in vacuo to obtain white solid 5,5′-bis(trimethylstannyl)-2,2′-bithiophene (1.29 g, 85% yield). ^{1}H NMR (400 MHz, CD_3OD) δ 7.28 – 7.24 (d, J = 3.30 Hz, 2H), 7.10 – 7.04 (d, J = 3.41 Hz, 2H), 0.40 – 0.32 (d, J = 11.94 Hz, 18H). ^{13}C NMR (101 MHz, CD_3OD): δ (ppm) 142.77, 136.85, 135.37, 124.51, -11.82.

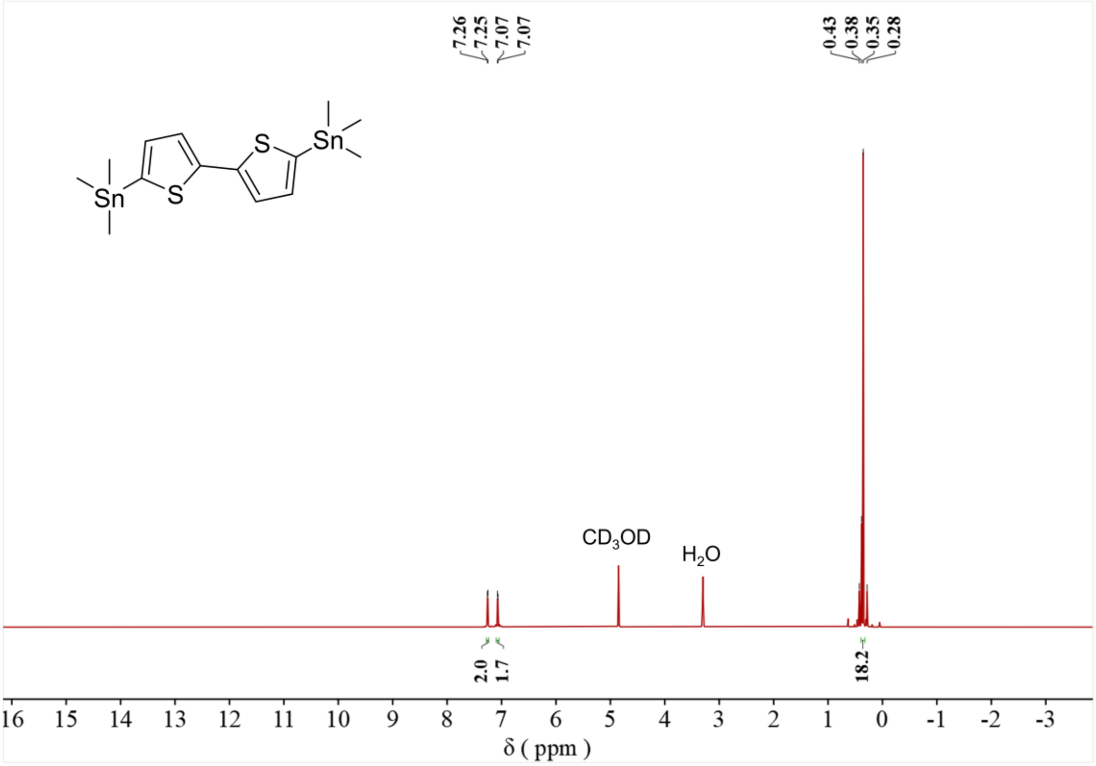

Supplementary Fig. 5 ^{1}H NMR spectrum of 2TSnMe3 in CD_3OD.

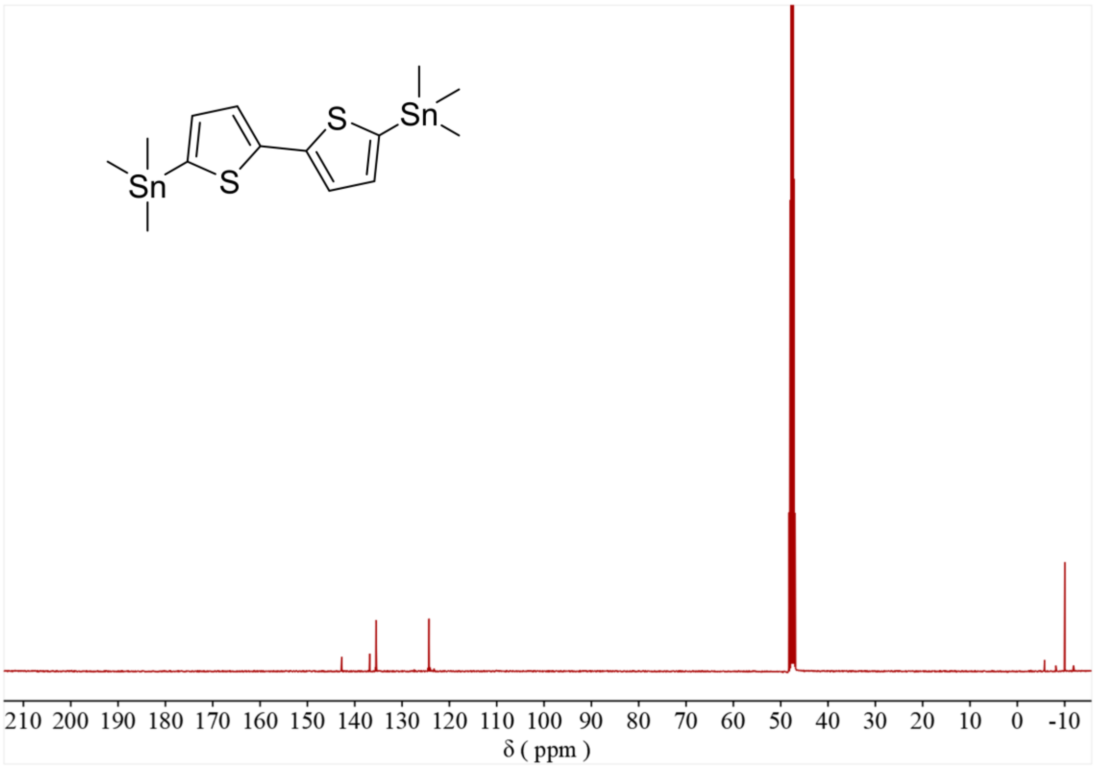

Supplementary Fig. 6 ^{13}C NMR spectrum of 2TSnMe$_3$ in CD$_3$OD.

Synthesis of 2TOSnMe$_3$. A solution of 3,3'-dimethoxy-2,2'-bithiophene (700 mg, 3.09 mmol, 1.00 eq.) in 150 mL of anhydrous tetrahydrofuran was cooled to -78 °C. To this solution, a 2.5 M solution of n-butyllithium in hexanes (5.0 mL, 13 mmol, 4.0 eq.) was subsequently added dropwise. The reaction was stirred for 30 min at -78 °C before being warmed to room temperature and stirred for an additional 30 min. The reaction mixture was cooled once again to -78 °C and trimethyltin chloride (2.59 g, 13.0 mmol, 4.20 eq.) was added in one portion. The reaction was allowed to warm to room temperature and was stirred overnight. The reaction mixture was diluted with diethyl ether and washed with water and brine before being dried over anhydrous sodium sulfate. Excess solvent was removed under reduced pressure. The reaction was purified by recrystallization from acetonitrile to yield the final product as pale-yellow crystals (1.22 g, 2.20 mmol, 71% yield). ^{1}H NMR (400 MHz, CDCl$_3$) δ 6.93 – 6.86 (s, 2H), 3.99 – 3.92 (s, 6H), 0.45 – 0.28 (m, 18H). ^{13}C NMR (101 MHz, CDCl$_3$) δ 154.85, 134.03, 123.36, 119.67, 59.04, -10.03.

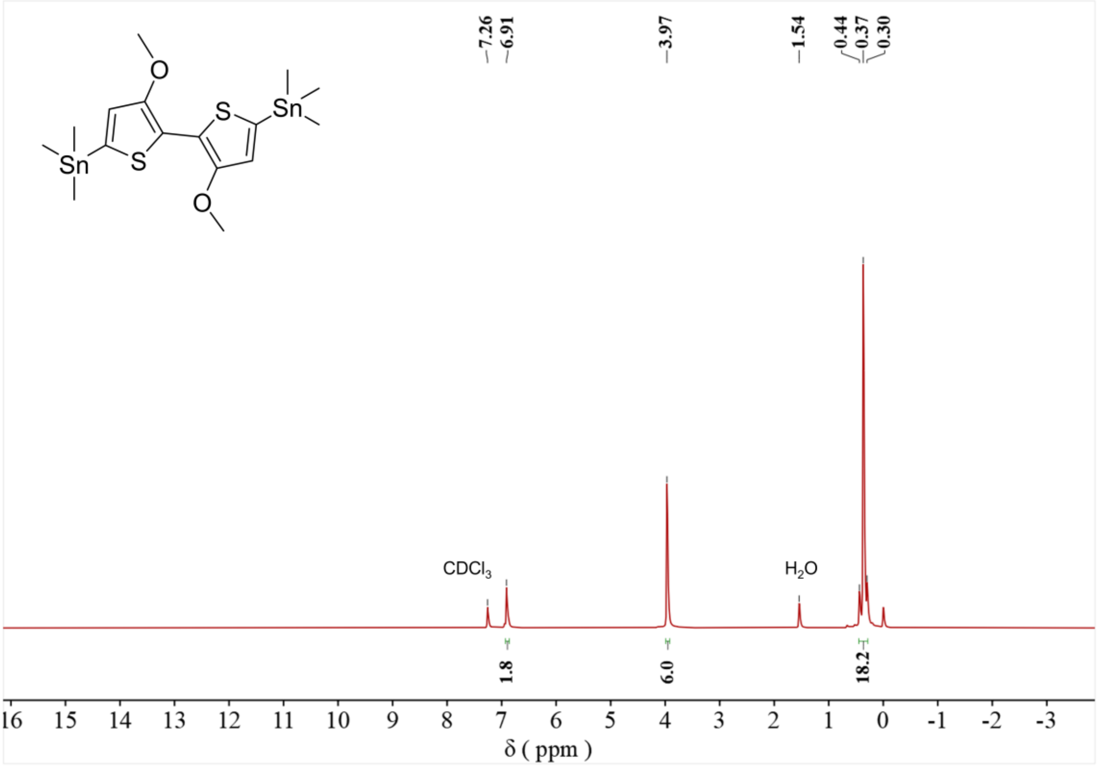

Supplementary Fig. 7 ^{1}H NMR spectrum of 2TOSnMe$_3$ in CDCl$_3$.

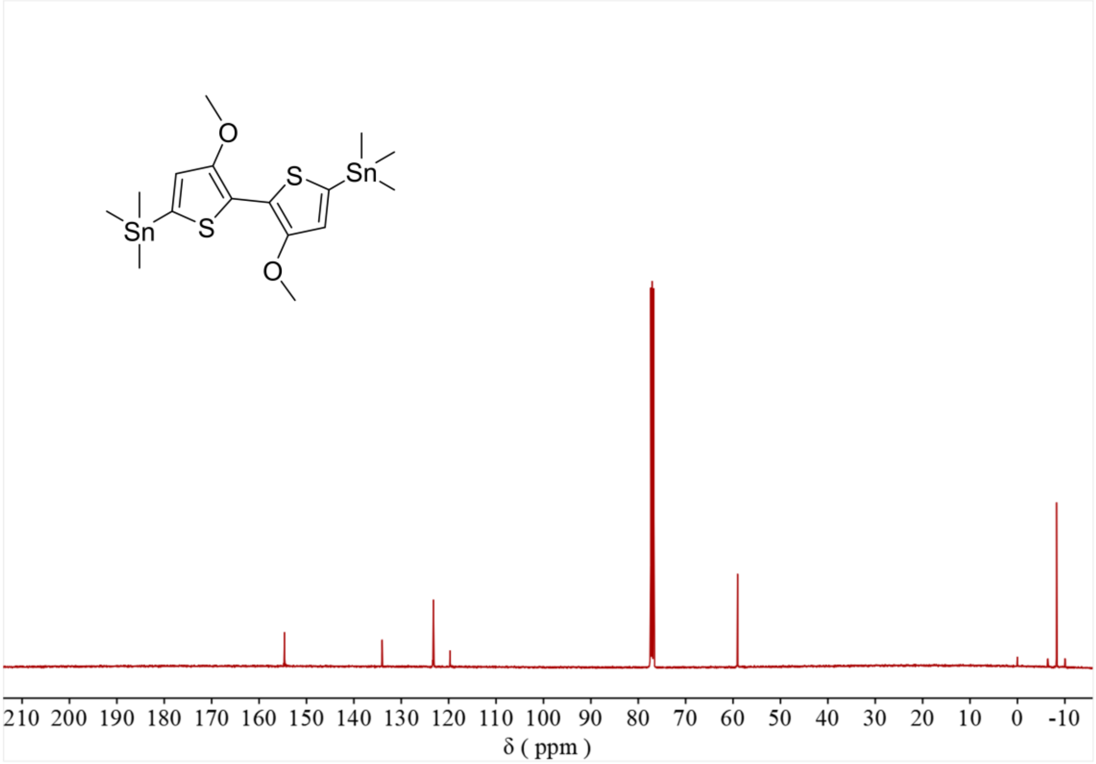

Supplementary Fig. 8 ^{13}C NMR spectrum of 2TOSnMe$_3$ in CDCl$_3$.

Synthesis of NDIalk. 2,6-Dibromonaphthalene-1,4,5,8-tetracarboxylic dianhydride (19.2 g, 45.1 mmol) and 2-octyldodecane-1-amine (39.5 g, 95 mmol) were refluxed overnight under argon in 600 mL of acetic acid. The solution was concentrated to about 50 mL, and 300 mL of water was added. The mixture was subsequently extracted with diethyl ether. The combined organic phases were washed with water and saturated with aqueous $NaHCO_3$-solution and dried over anhydrous Na_2SO_4. The yellow soild product was purified by silica gel column chromatography with toluene/hexane 1:1 (v:v) as eluent followed by recrystallization from isopropanol (20.0 g, 20.3 mmol, 45% yield). ^{1}H NMR (400 MHz, $CDCl_3$) δ 9.01 – 8.97 (s, 2H), 4.18 – 4.11 (t, *J* = 6.39 Hz, 4H), 2.04 – 1.92 (m, 2H), 1.40 – 1.20 (m, 64H), 0.90 – 0.82 (m, *J* = 6.10 Hz, 12H). ^{13}C NMR (101 MHz, $CDCl_3$) δ 161.12, 139.17, 128.38, 127.76, 125.30, 124.10, 45.46, 36.46 , 31.91, 31.56, 30.03, 29 .61, 29.33, 26.34, 22.68, 14.12.

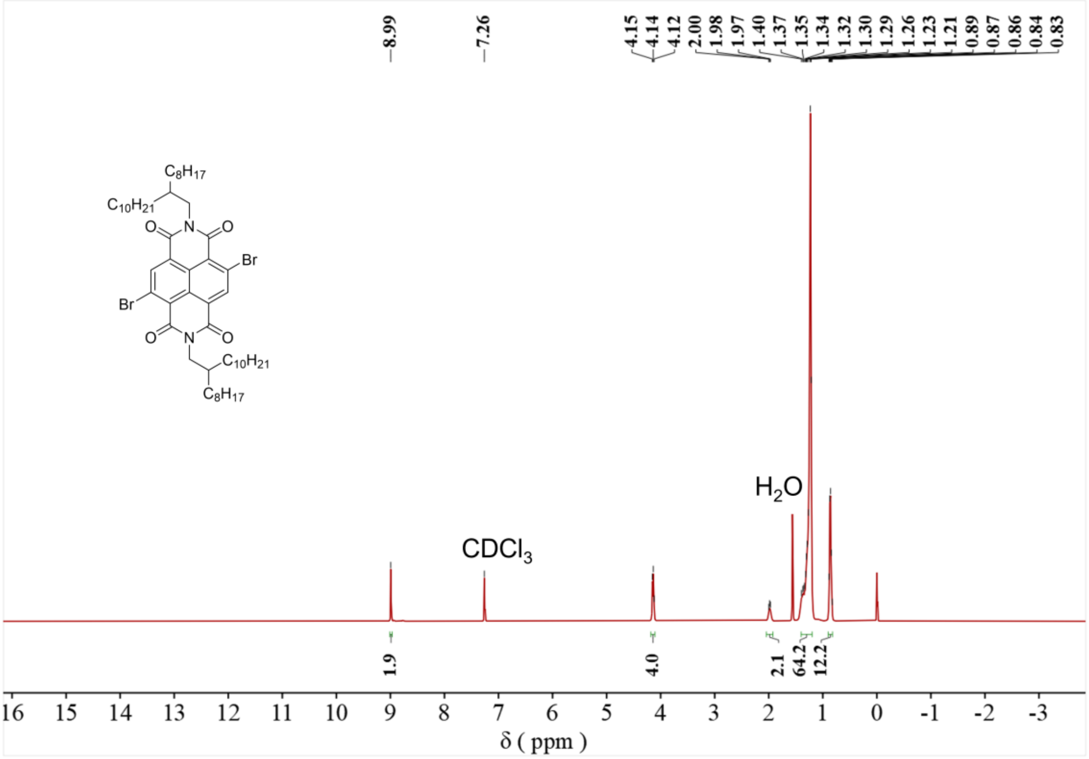

Supplementary Fig. 9 ^{1}H NMR spectrum of NDIalk in $CDCl_3$.

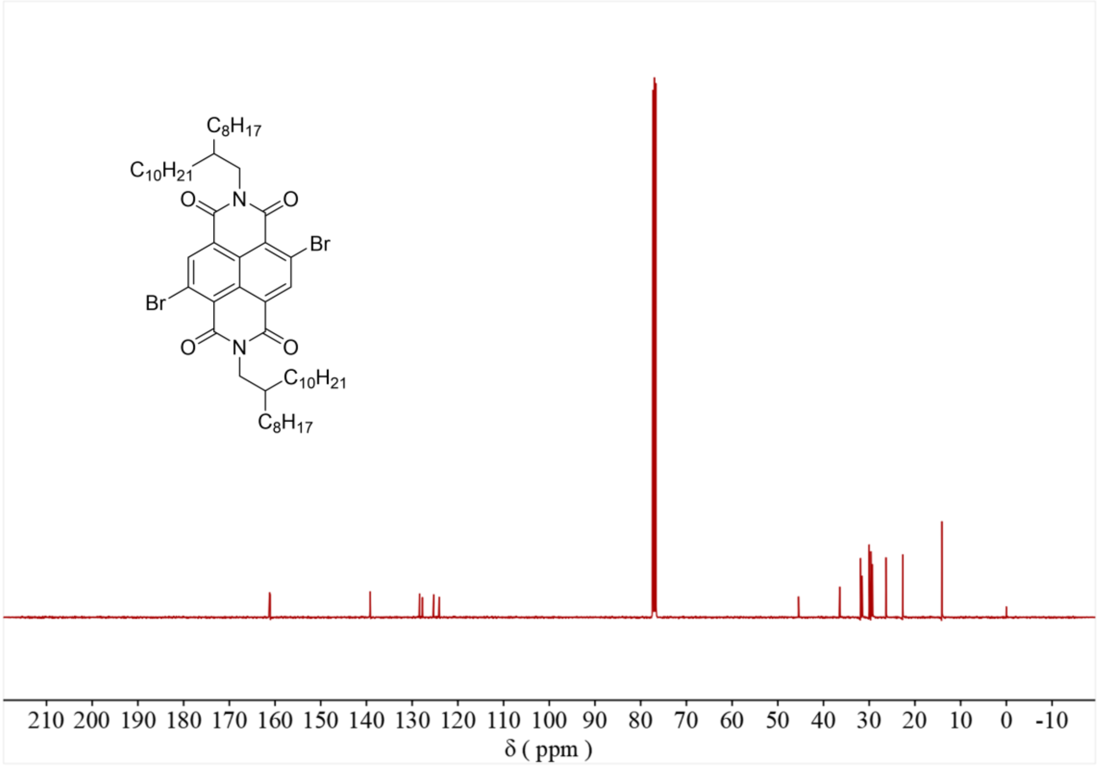

Supplementary Fig. 10 ^{13}C NMR spectrum of NDIalk in $CDCl_3$.

Synthesis of NDIgly. A 100 mL two-neck RBF was dried and purged with argon. 2,5,8,11,14,17,20-heptaoxadocosan-22- amine (990 mg, 2.91 mmol), anhydrous zinc acetate (535 mg, 2.91 mmol) and 2,6-dibromonaphthalene-1,4,5,8-tetracarboxylic dianhydride (620 mg, 1.45 mmol) were dissolved in 50 mL of anhydrous xylene. The reaction mixture was heated to 130 °C for 3 h and the conversion was monitored by NMR (addition of further amine if necessary). 100 mL of chloroform was added and the organic phase was washed with deionized water (3 x 100 mL) and dried over $MgSO_4$. The solvent was removed and the yellow solid was purified by column chromatography (silica gel, ethyl acetate: MeOH = 10: 1, v:v). A yellow solid (NDIgly) was obtained (435 mg, 28% yield). ^{1}H NMR (400 MHz, CD_3OD) δ 8.86 – 8.78 (s, 2H), 4.42 – 4.36 (t, *J* = 5.98 Hz, 4H), 3.84 – 3.77 (t, *J* = 6.03 Hz, 4H), 3.69 – 3.64 (dd, *J* = 3.03, 6.30 Hz, 4H), 3.61 – 3.51 (m, 40H), 3.36 – 3.32 (s, 6H). ^{13}C NMR (100 MHz, CD_3OD) δ 160.95, 160.72, 137.89, 127.71, 127.13, 125.57, 124.16, 71.58, 70.15, 69.96, 67.21, 57.71, 39.67.

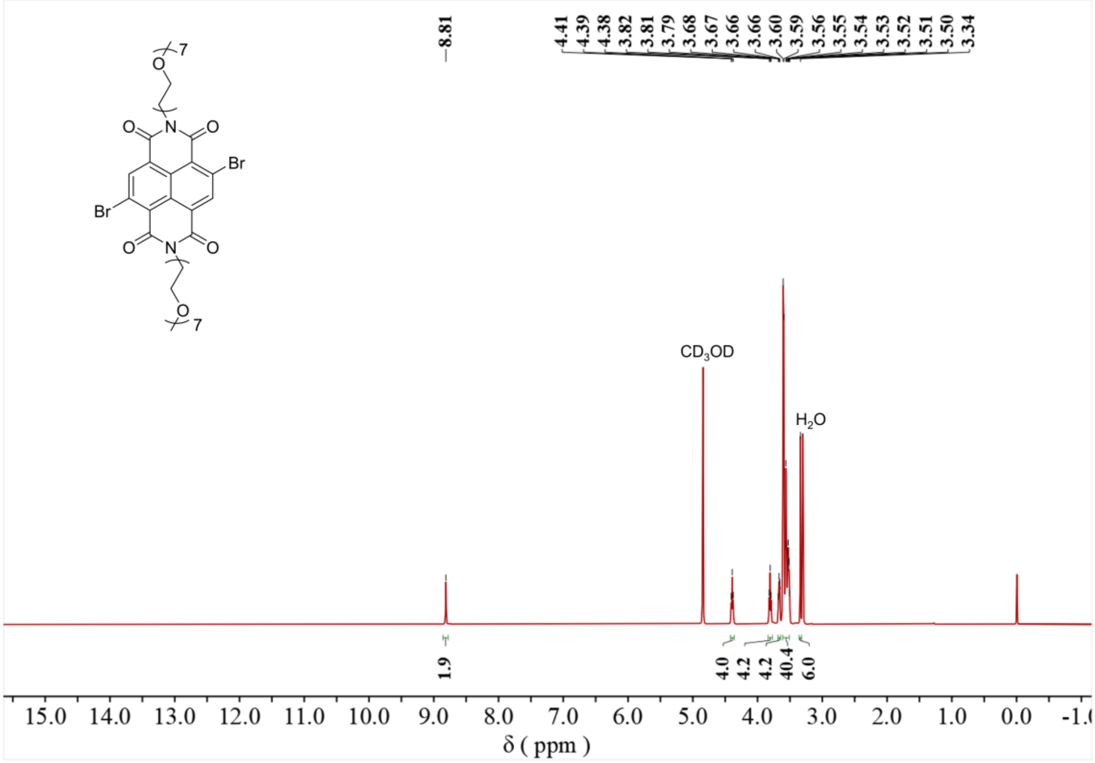

Supplementary Fig. 11 ^{1}H NMR spectrum of NDIgly in CD_3OD.

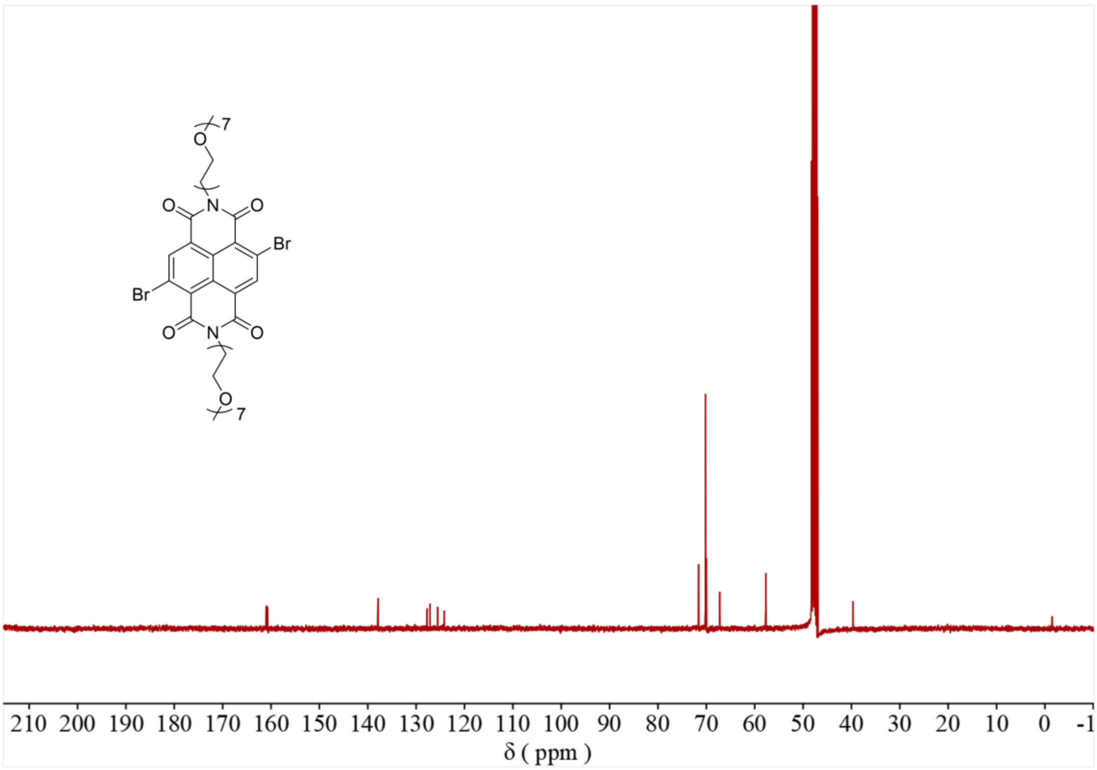

Supplementary Fig. 12 ^{13}C NMR spectrum of NDIgly in CD_3OD.

Synthesis of NTalk polymer.[11] The monomer (*N,N'*-bis(7-glycol)-2,6-dibromonaphthalene-1,4,5,8-bis(dicarboximide) (NDIgly) (106.8 mg, 0.1 mmol) and 5,5'-bis(trimethylstannyl)-2,2'-bithiophene ($2TSnMe_3$) (49.1 mg, 0.1 mmol) were dissolved in 1.5 mL of anhydrous and degassed chlorobenzene. $Pd_2(dba)_3$ (2 mol%) and $P(o\text{-}tol)_3$ (8 mol%) were added and the vial was sealed and heated to 135 °C for 5 h. After the polymerization was finished, the end-capping procedure was carried out. Then, the reaction mixture was cooled to room temperature and the reaction mixture was precipitated in ethyl acetate followed by the addition of hexane. The solid was collected in a glass-fiber thimble and Soxhlet extraction was carried out with hexane, ethyl acetate, MeOH, acetone and chloroform. The polymers dissolved in hot chloroform (40 ^{O}C) and the solvent was removed under reduced pressure. Finally, the polymers were precipitated in ethyl acetate and the dark blue solids were dried under high vacuum for 12 h. Polymer NTalk was obtained as a dark blue solid (108.9 mg). GPC (THF, 40 °C): Mn = 16.2 kDa, PDI = 1.292.

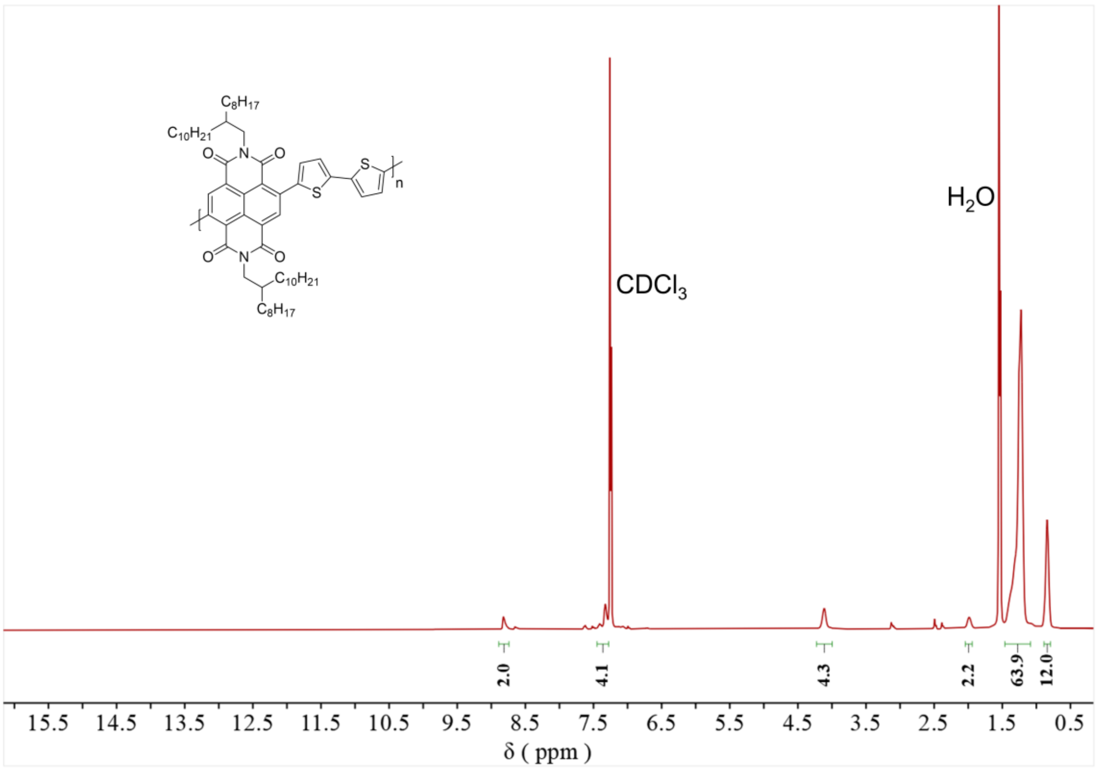

Supplementary Fig. 13 ^{1}H NMR spectrum of NTalk in $CDCl_3$.

Synthesis of NTOalk polymer.[11] The monomer NDIalk (98.5 mg, 0.1 mmol) and $2TOSnMe_3$ (55.2 mg, 0.1 mmol) were dissolved in 1.5 mL of anhydrous, degassed chlorobenzene. $Pd_2(dba)_3$ (2 mol%) and $P(o\text{-}tol)_3$ (8 mol%) were added and the vial was sealed and heated to 135 °C for 4 h. After the polymerization was finished, the end-capping procedure was carried out. Then, the reaction mixture was cooled to room temperature and the reaction mixture was precipitated in ethyl acetate followed by the addition of hexane. The solid was collected in a glass-fiber thimble and Soxhlet extraction was carried out with hexane, ethyl acetate, MeOH, acetone and chloroform.

The polymers were dissolved in hot chloroform (40 ^{O}C) and the solvent was removed under reduced pressure. Finally, the polymers were precipitated in ethyl acetate and the dark green solids were dried under high vacuum for 12 h. Polymer NTOalk was obtained as a dark blue solid (115.6 mg). GPC (THF, 40 °C): Mn = 16.8 kDa, PDI = 1.226.

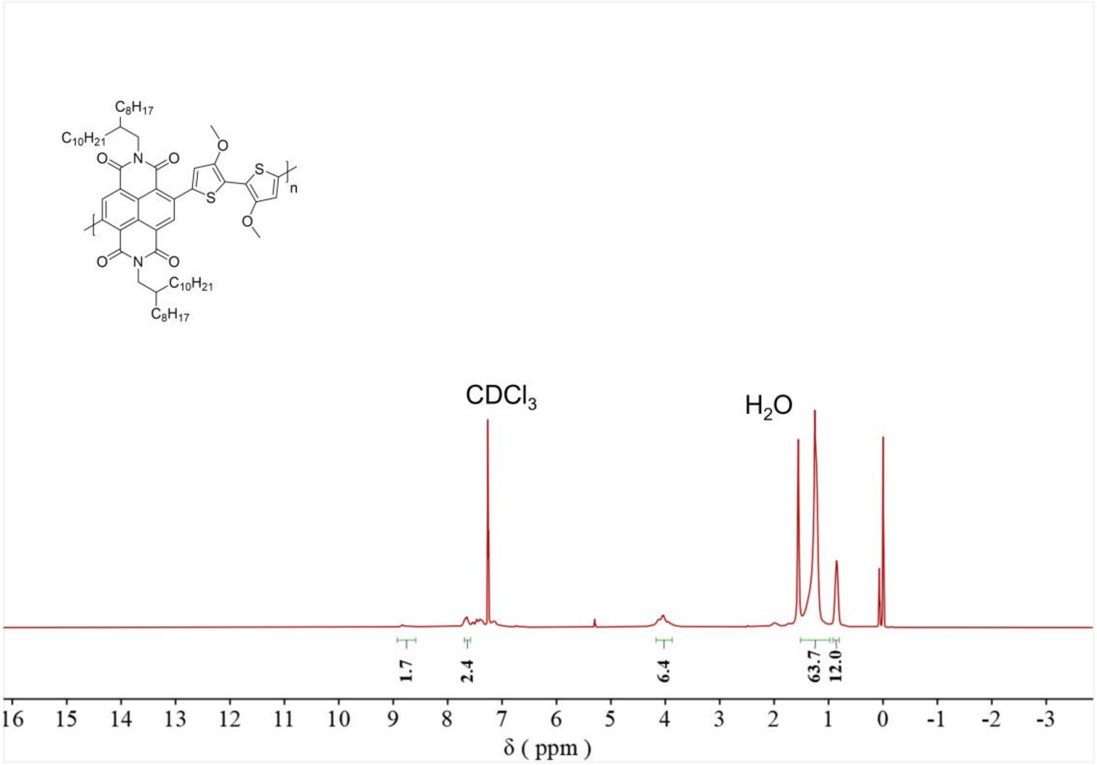

Supplementary Fig. 14 ^{1}H NMR spectrum of NTOalk measured in $CDCl_3$.

Synthesis of NTgly polymer.[11] The monomer NDIgly (106.8 mg, 0.1 mmol) and $2TSnMe_3$ (49.1 mg, 0.1 mmol) were dissolved in 1.5 mL of anhydrous, degassed chlorobenzene. $Pd_2(dba)_3$ (2 mol%) and $P(o\text{-}tol)_3$ (8 mol%) were added and the vial was sealed and heated to 135 °C for 8 h. After the polymerization was finished, the end-capping procedure was carried out. Then, the reaction mixture was cooled to room temperature and the reaction mixture was precipitated in ethyl acetate followed by the addition of hexane. The solid was collected in a glass-fiber thimble and Soxhlet extraction was carried out with hexane, ethyl acetate, MeOH, acetone and chloroform. The polymers dissolved in hot chloroform (40 ^{O}C) and the solvent was removed under reduced pressure. Finally, the polymers were precipitated in ethyl acetate and the dark blue solids were dried under high vacuum for 12 h. NTgly was obtained as a dark blue solid (98.7 mg). GPC (THF, 40 °C): Mn = 15.9 kDa, PDI = 1.215.

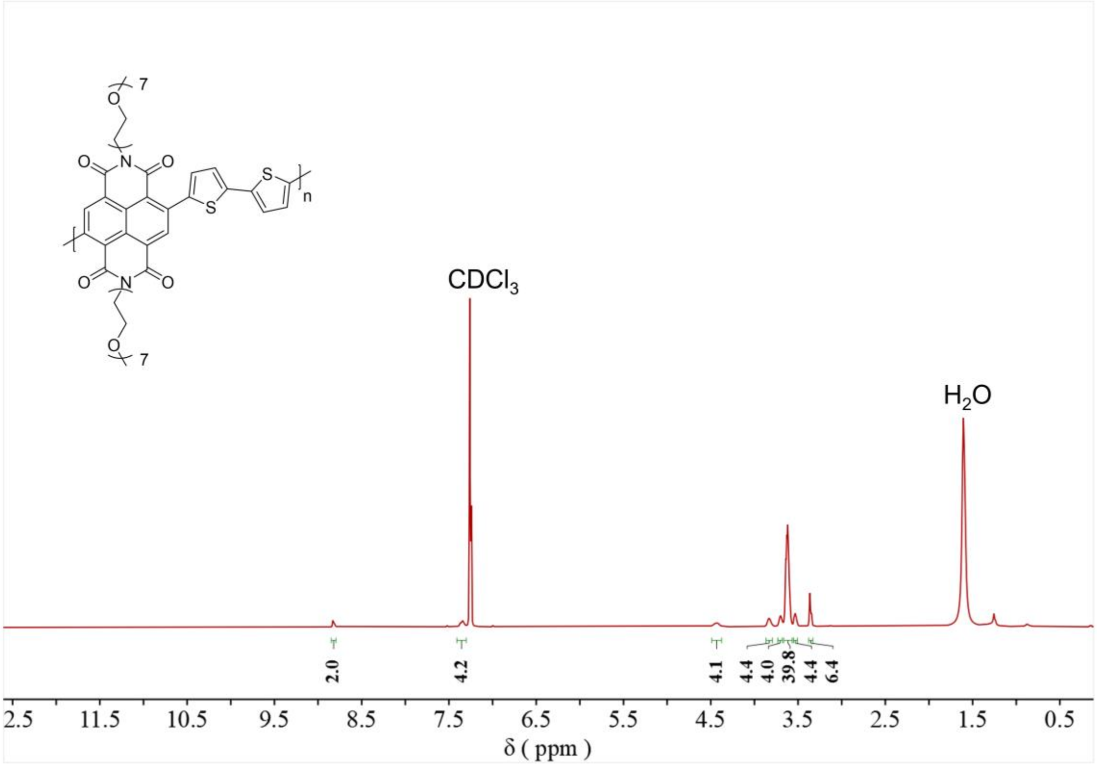

Supplementary Fig. 15 ^{1}H NMR spectrum of NTgly measured in $CDCl_3$.

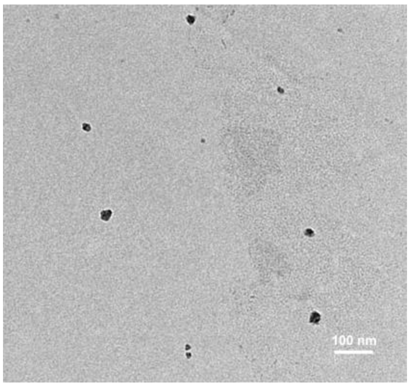

Supplementary Fig. 16 TEM image of NTOalk NPs.

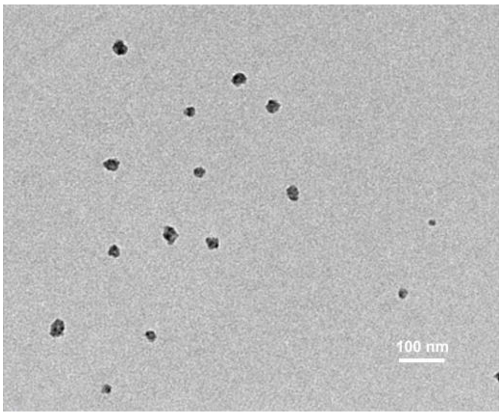

Supplementary Fig. 17 TEM image of NTalk NPs.

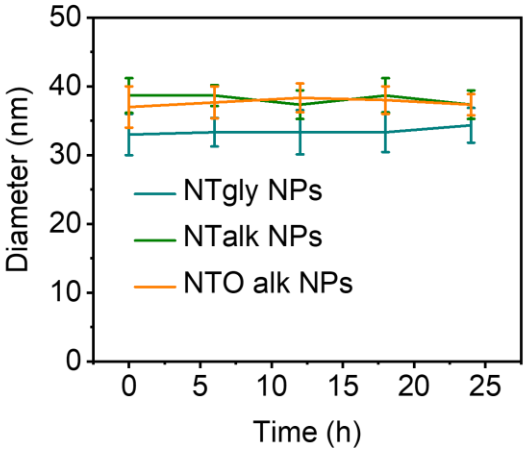

Supplementary Fig. 18 The structural stability of NTOalk NPs, NTalk NPs and NTgly NPs during storage in PBS buffer (pH 7.4) containing 10% FBS at 37 ºC for 24 h.

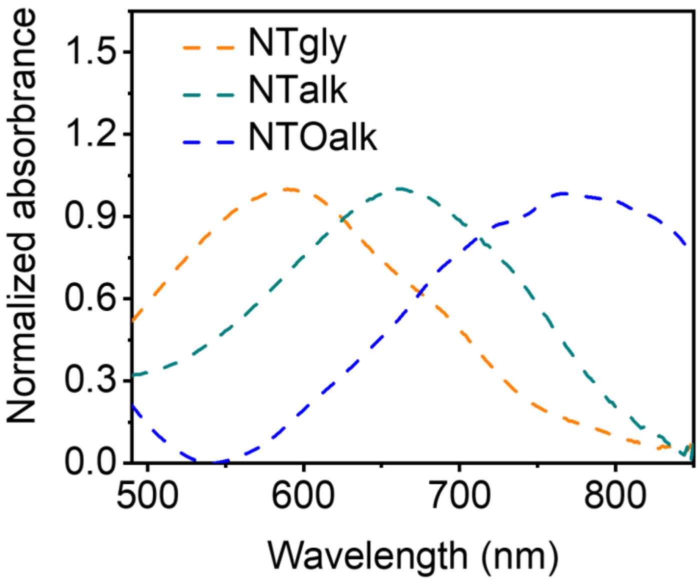

Supplementary Fig. 19 Normalized absorption spectra of polymer PSs in dichloromethane.

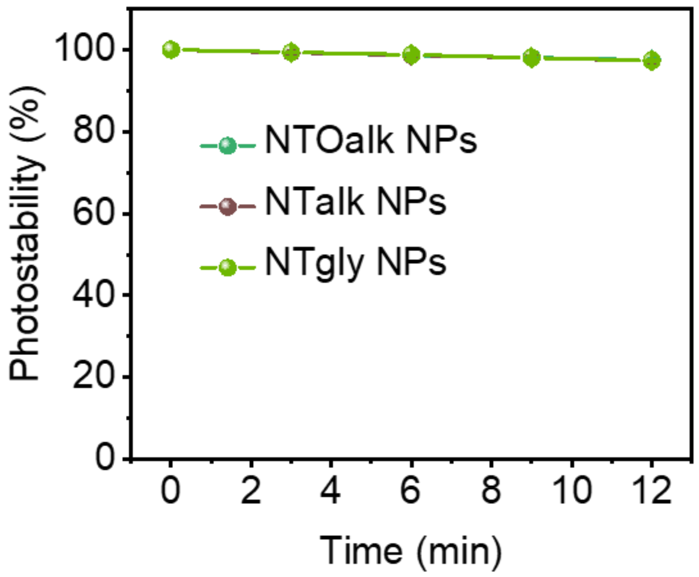

Supplementary Fig. 20 The photostability of three PSs was first evaluated under 808 nm with a power of 330 mW cm^{-2} for 10 min in PBS (pH = 7) containing 10% FBS.

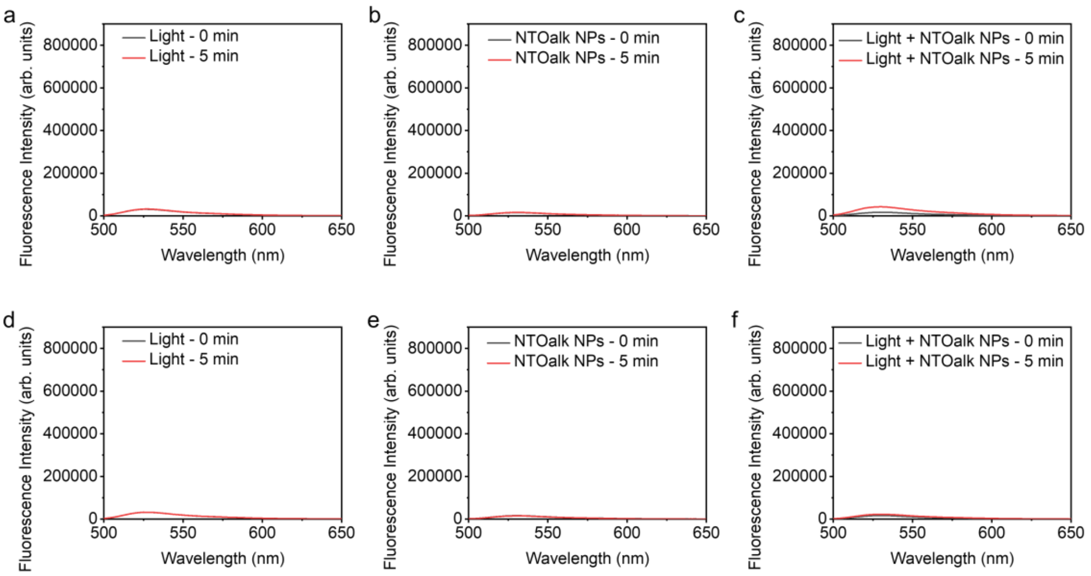

Supplementary Fig. 21 808 nm laser-driven generation of $\bullet O_2^-$ for NTOalk NPs using the DHR 123 indicator. Fluorescence spectra changes of DHR 123 (10.0 μM) for PBS under the 808 nm laser irradiation in (a) normal and (d) hypoxic conditions within 5 min. Fluorescence spectra changes of DHR 123 for NTOalk NPs in (b) normal and (e) hypoxic conditions within 5 min. Fluorescence spectra changes of DHR 123 induced by NTOalk NPs under the 808 nm laser irradiation in (c) normal and (f) hypoxic conditions within 5 min. [DHR 123] = 10.0 μM and [NPs] = 5 μg/mL. The power density of 808 nm is 15 mW cm^{-2}.

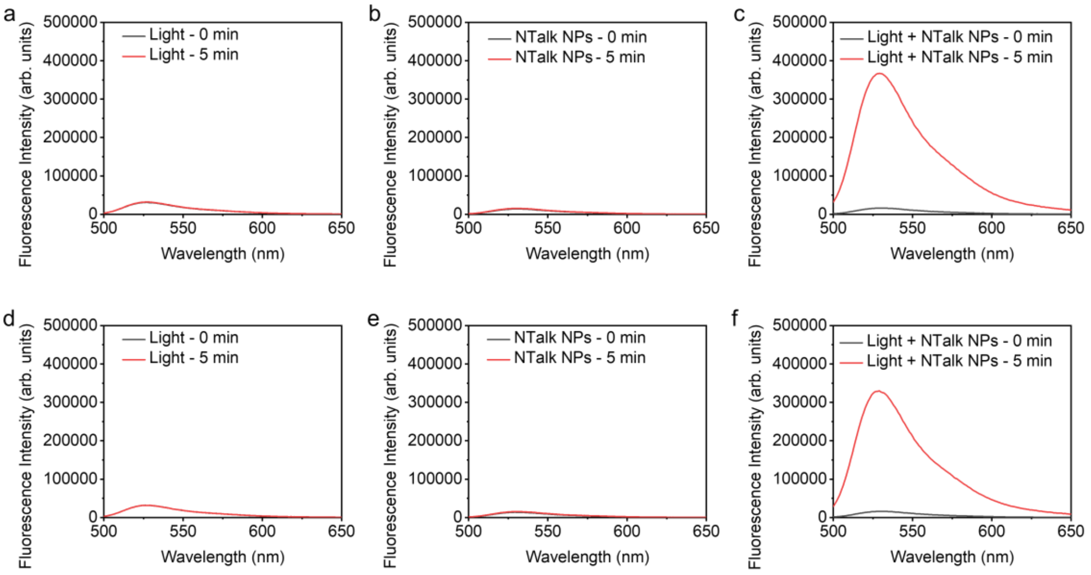

Supplementary Fig. 22 808 nm laser-driven generation of $\bullet O_2^-$ for NTalk NPs using the DHR 123 indicator. Fluorescence spectra changes of DHR 123 (10.0 μM) for PBS under the 808 nm laser irradiation in (a) normal and (d) hypoxic conditions within 5 min. Fluorescence spectra changes of DHR 123 for NTalk NPs in (b) normal and (e) hypoxic conditions within 5 min. Fluorescence spectra changes of DHR 123 induced by NTalk NPs under the 808 nm laser irradiation in (c) normal and (f) hypoxic conditions within 5 min. [DHR 123] = 10.0 μM and [NPs] = 5 μg/mL. The power

density of 808 nm is 15 mW cm^{-2}.

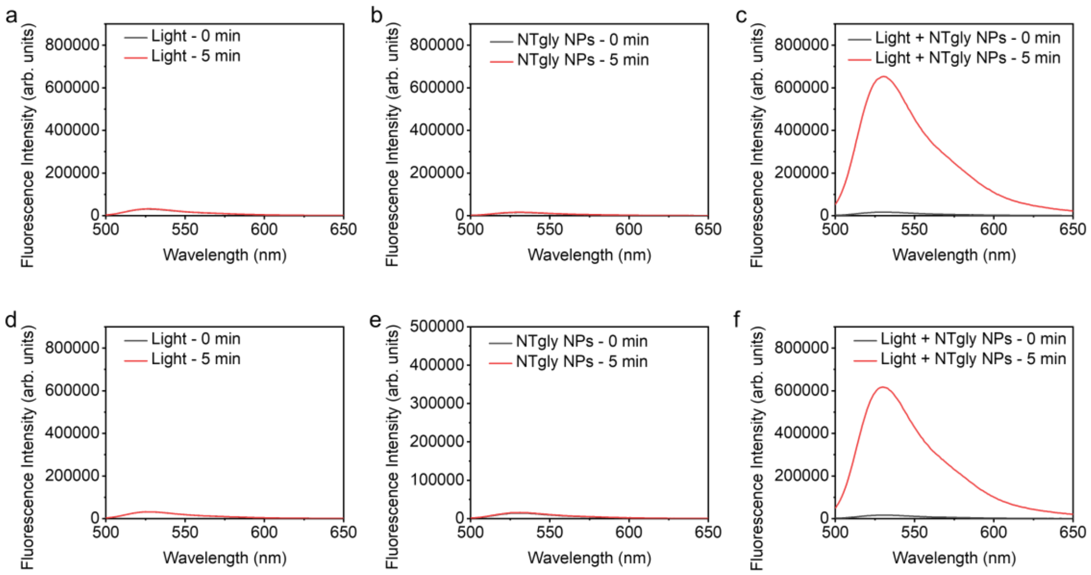

Supplementary Fig. 23 808 nm laser-driven generation of $\bullet O_2^-$ for NTgly NPs using the DHR 123 indicator. Fluorescence spectra changes of DHR 123 for PBS under the 808 nm laser irradiation in (a) normal and (d) hypoxic conditions within 5 min. Fluorescence spectra changes of DHR 123 for NTgly NPs in (b) normal and (e) hypoxic conditions within 5 min. Fluorescence spectra changes of DHR 123 induced by NTgly NPs under the 808 nm laser irradiation in (c) normal and (f) hypoxic conditions within 5 min. [DHR 123] = 10.0 μM and [NPs] = 5 μg/mL. The power density of 808 nm is 15 mW cm^{-2}.

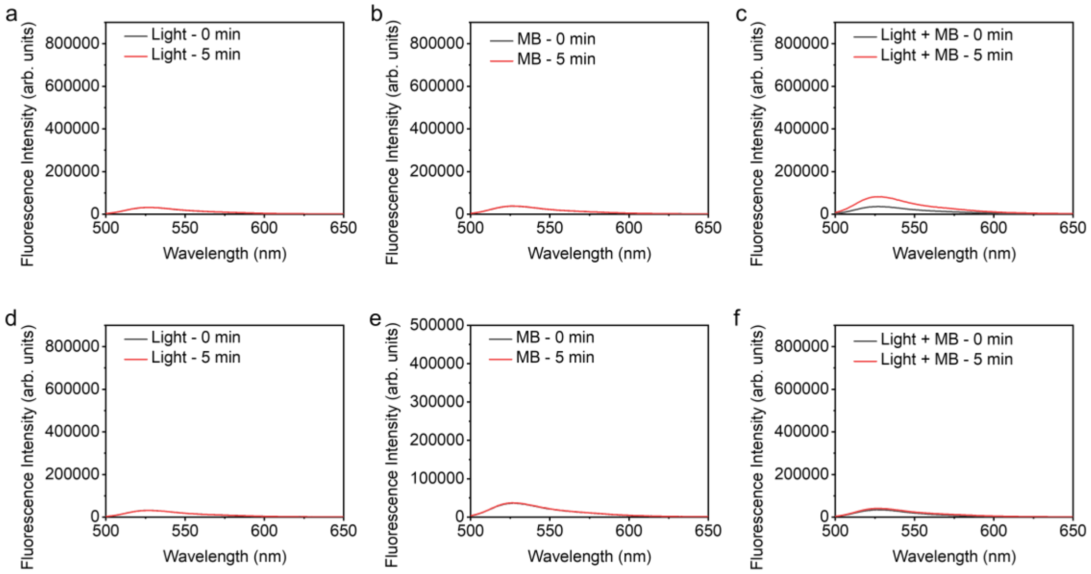

Supplementary Fig. 24 635 nm laser-driven generation of $\bullet O_2^-$ for MB using the DHR 123 indicator. Fluorescence spectra changes of DHR 123 for PBS under the 635 nm laser irradiation in (a) normal and (d) hypoxic conditions within 5 min. Fluorescence spectra changes of DHR 123 for MB in (b) normal and (e) hypoxic conditions within 5 min. Fluorescence spectra changes of DHR 123 induced by MB under the 635 nm

laser irradiation in (c) normal and (f) hypoxic conditions within 5 min. [DHR 123] = 10.0 µM, and [MB] = 0.1 µg/mL. The molal concentration of MB and NTgly is equal. The power density of 635 nm is 15 mW cm^{-2}.

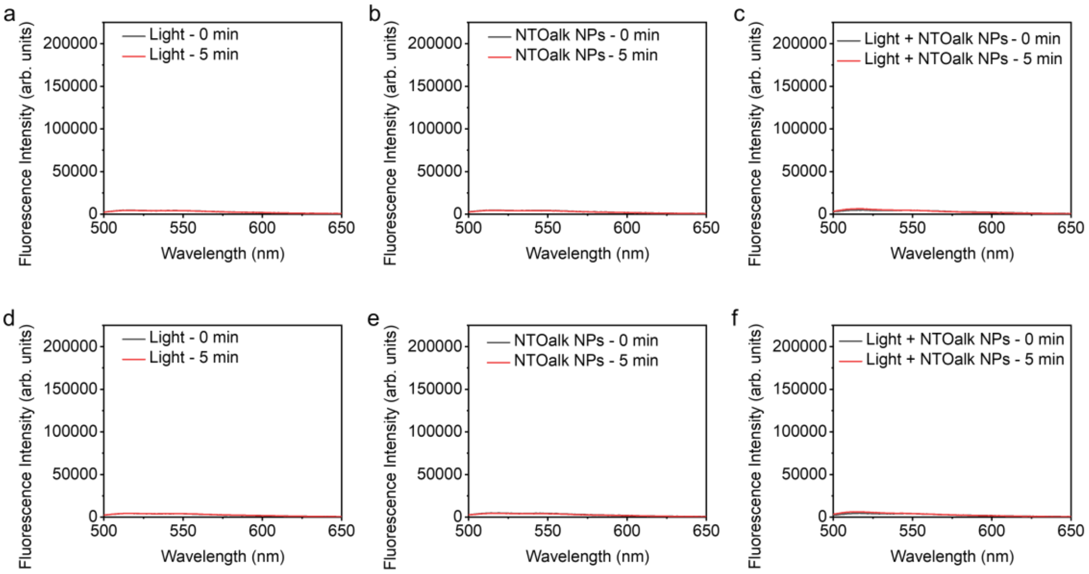

Supplementary Fig. 25 808 nm laser-driven generation of •OH for NTOalk NPs using the HPF indicator. Fluorescence spectra changes of HPF for PBS under the 808 nm laser irradiation in (a) normal and (d) hypoxic conditions within 5 min. Fluorescence spectra changes of HPF for NTOalk NPs in (b) normal and (e) hypoxic conditions within 5 min. Fluorescence spectra changes of HPF induced by NTOalk NPs under the 808 nm laser irradiation in (c) normal and (f) hypoxic conditions within 5 min. [HPF] = 10.0 µM and [NPs] = 5 µg/mL. The power density of 808 nm is 15 mW cm^{-2}.

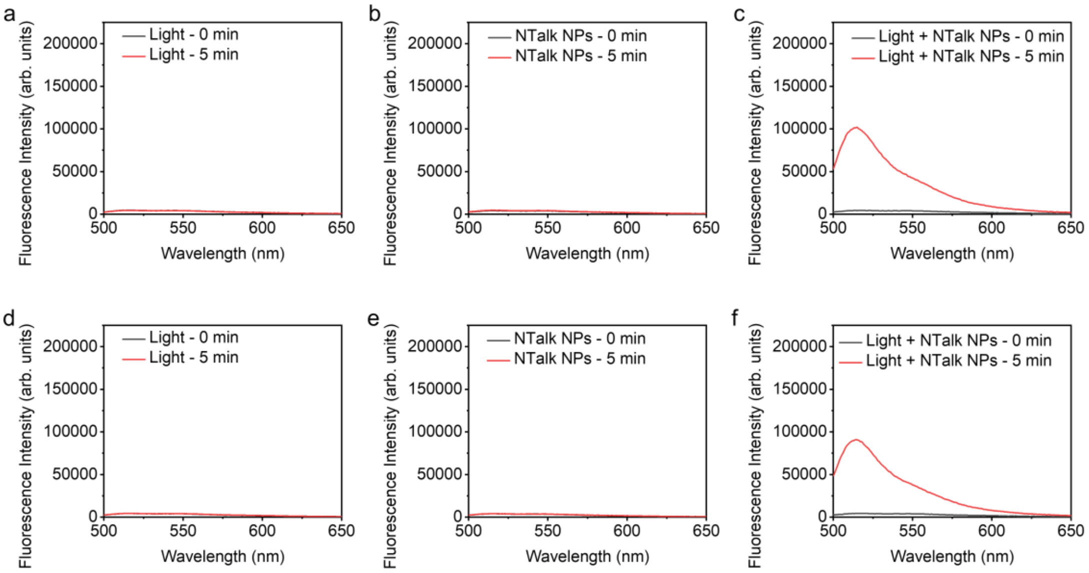

Supplementary Fig. 26 808 nm laser-driven generation of •OH for NTalk NPs using the HPF indicator. Fluorescence spectra changes of HPF for PBS under the 808 nm laser irradiation in (a) normal and (d) hypoxic conditions within 5 min. Fluorescence spectra changes of HPF for NTalk NPs in (b) normal and (e) hypoxic conditions

within 5 min. Fluorescence spectra changes of HPF induced by NTalk NPs under the 808 nm laser irradiation in (c) normal and (f) hypoxic conditions within 5 min. [HPF] = 10.0 μM and [NPs] = 5 μg/Ml. The power density of 808 nm is 15 mW cm^{-2}.

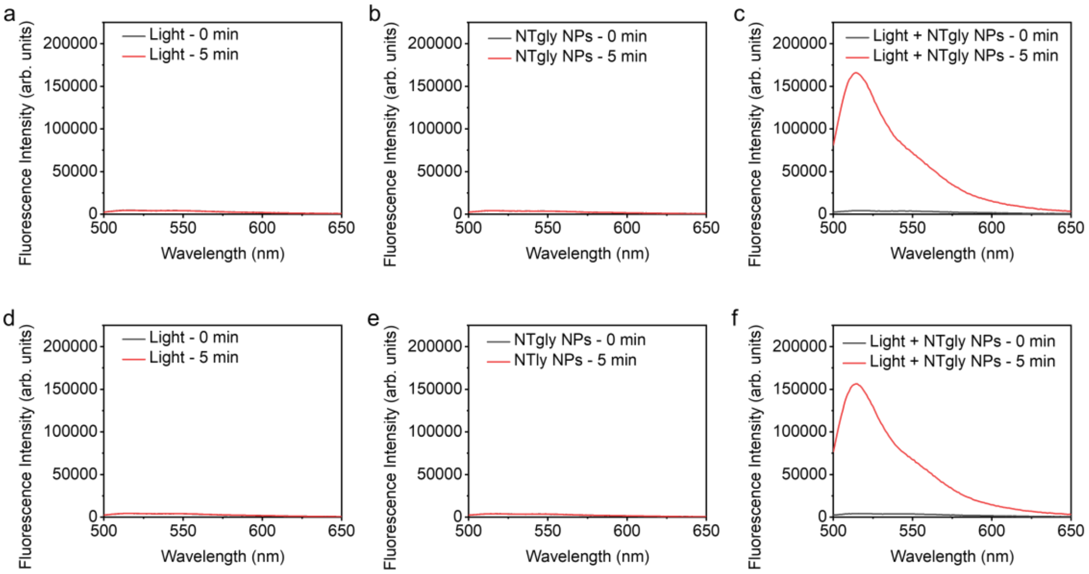

Supplementary Fig. 27 808 nm laser-driven generation of •OH for NTgly NPs using the HPF indicator. Fluorescence spectra changes of HPF for PBS under the 808 nm laser irradiation in (a) normal and (d) hypoxic conditions within 5 min. Fluorescence spectra changes of HPF for NTgly NPs in (b) normal and (e) hypoxic conditions within 5 min. Fluorescence spectra changes of HPF induced by NTgly NPs under the 808 nm laser irradiation in (c) normal and (f) hypoxic conditions within 5 min. [HPF] = 10.0 μM and [NPs] = 5 μg/mL. The power density of 808 nm is 15 mW cm^{-2}.

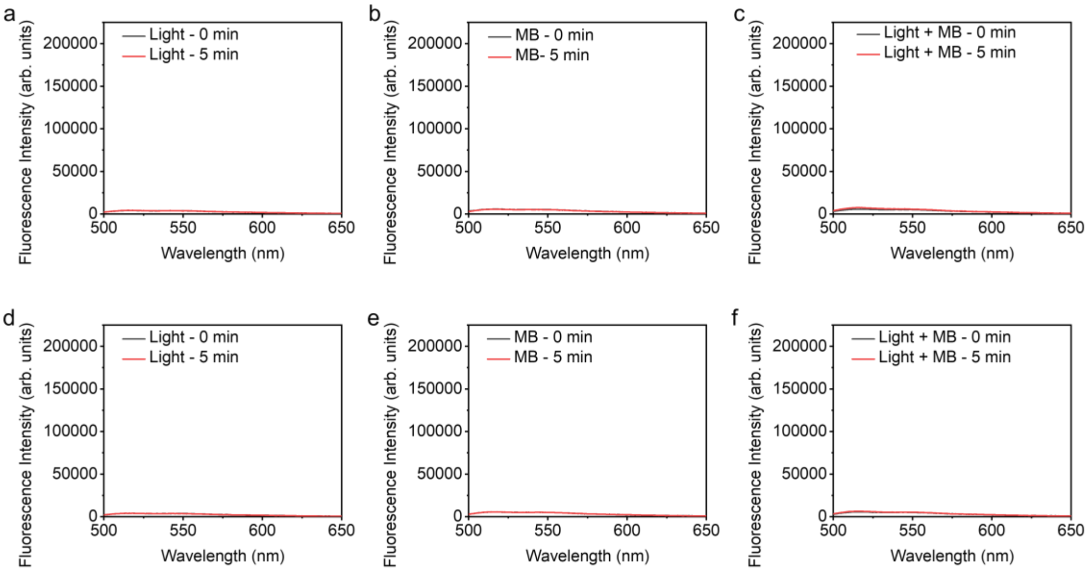

Supplementary Fig. 28 635 nm laser-driven generation of •OH for MB using the HPF indicator. Fluorescence spectra changes of HPF for PBS under the 635 nm laser irradiation in (a) normal and (d) hypoxic conditions within 5 min. Fluorescence spectra changes of HPF for MB in (b) normal and (e) hypoxic conditions within 5 min.

Fluorescence spectra changes of HPF induced by MB under the 635 nm laser irradiation in (c) normal and (f) hypoxic conditions within 5 min. [HPF] = 10.0 μM and [NPs] = 5 μg/mL, [MB] = 0.1 μg/mL. The molal concentration of MB and NTgly is equal. The power density of 635 nm is 15 mW cm^{-2}.

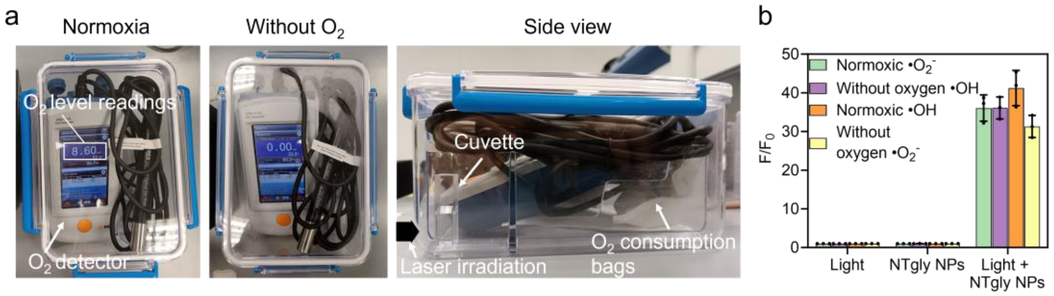

Supplementary Fig. 29 (a) The AnaeroPack™-Anaero Anaerobic Gas Generator within a sealed transparent chamber. (b) The fluorescence intensity changes of •O_2^- probe (DHR 123) at 525 nm and •OH probe (HPF) at 515 nm for NTgly NPs (5 μg mL-1) under 808 nm irradiation at 15 mW cm^{-2} for 5 min.

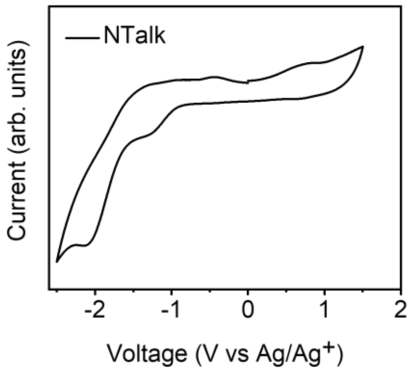

Supplementary Fig. 30 Cyclic voltammetry curve of NTalk. The electrochemical cyclic voltammetry was recorded on an AUTOAB electrochemical analyzer with glassy carbon, Pt wire, and Ag/AgCl as working, counter and reference electrodes, respectively, in 0.10 M $(n\text{-}Bu)_4NPF_6$ acetonitrile at a scan rate of 50 mV/s.

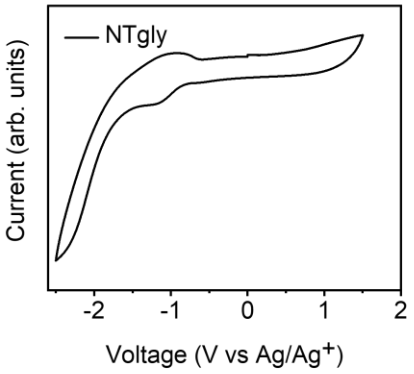

Supplementary Fig. 31 Cyclic voltammetry curve of NTgly. The electrochemical cyclic voltammetry was recorded on an AUTOAB electrochemical analyzer with glassy carbon, Pt wire, and Ag/AgCl as working, counter and reference electrodes, respectively, in 0.10 M (n-Bu)$_4$NPF$_6$ acetonitrile at a scan rate of 50 mV/s.

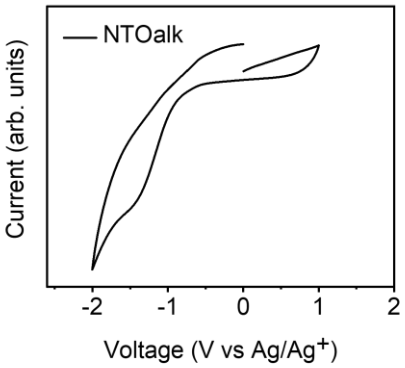

Supplementary Fig. 32 Cyclic voltammetry curve of NTalk. The electrochemical cyclic voltammetry was recorded on an AUTOAB electrochemical analyzer with glassy carbon, Pt wire, and Ag/AgCl as working, counter and reference electrodes, respectively, in 0.10 M (n-Bu)$_4$NPF$_6$ acetonitrile at a scan rate of 50 mV/s.

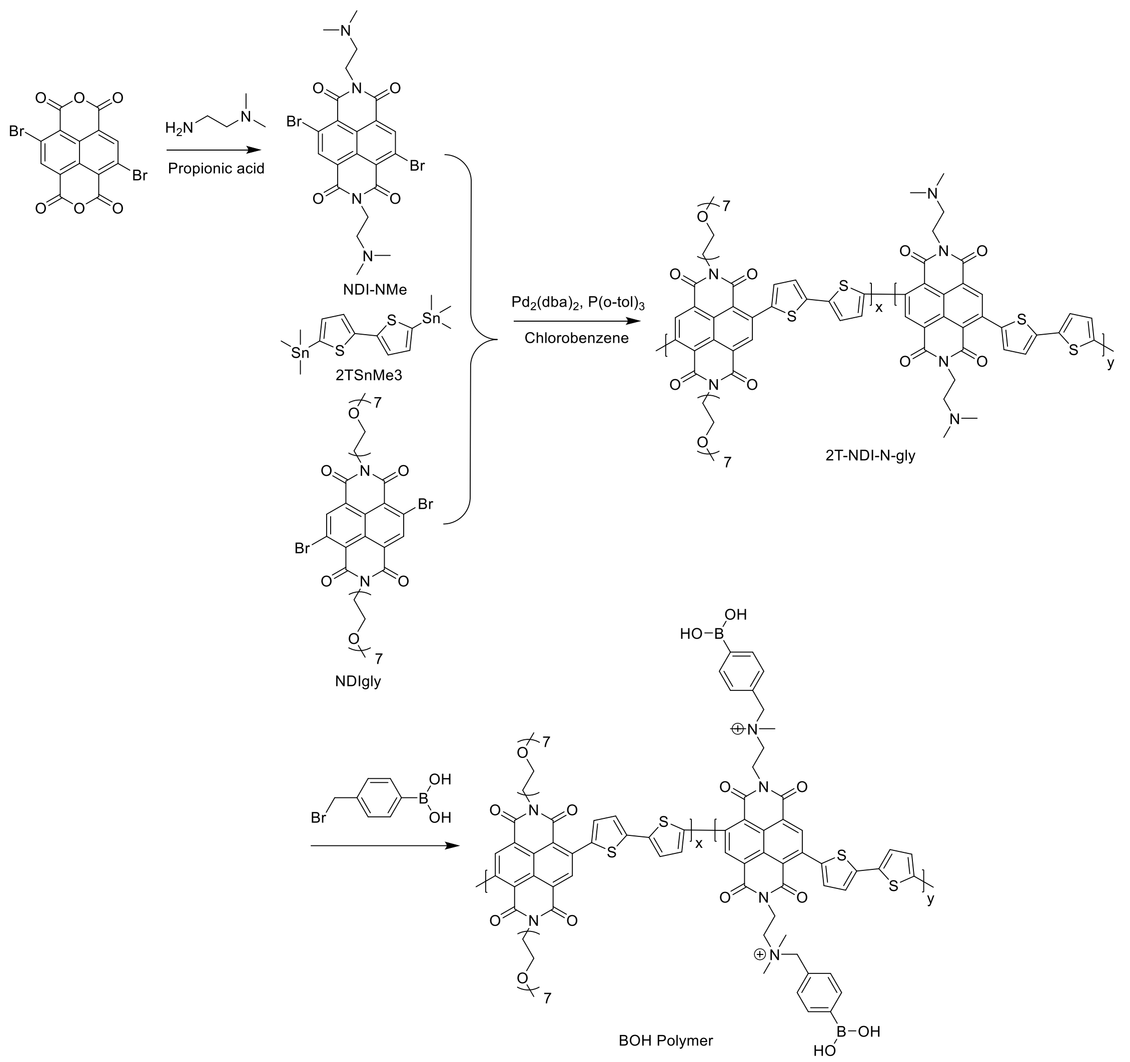

Supplementary Fig. 33 Overview of the synthesis of BOH polymer.

Synthesis of 2T-NDI-N-gly polymer.[12] NDI-NMe was prepared according to the literature procedures.[13] The monomer NDIgly (74.8 mg, 0.07 mmol), NDI-NMe (16.7 mg, 0.03 mmol) and $2TSnMe_3$ (49.1 mg, 0.1 mmol) were dissolved in 1.5 mL of anhydrous and degassed chlorobenzene. $Pd_2(dba)_3$ (2 mol%) and $P(o\text{-}tol)_3$ (8 mol%) were added and the vial was sealed and heated to 135 °C for 3 h. After the polymerization was finished, the end-capping procedure was carried out. Then, the reaction mixture was cooled to room temperature and the reaction mixture was precipitated in ethyl acetate followed by the addition of hexane. The solid was collected in a glass-fibre thimble and Soxhlet extraction was carried out with hexane, ethyl acetate, MeOH, acetone and chloroform. The polymers were dissolved in hot chloroform (40 ^{O}C) and the solvent was removed under reduced pressure. Finally, the polymers were precipitated in ethyl acetate and the dark blue solids were dried to obtain 108.6 mg. Polymer 2T-NDI-N-gly (x : y = 6:3.1) was obtained as a dark blue solid. GPC (THF, 40 °C): Mn = 6.8 kDa, PDI = 2.05.

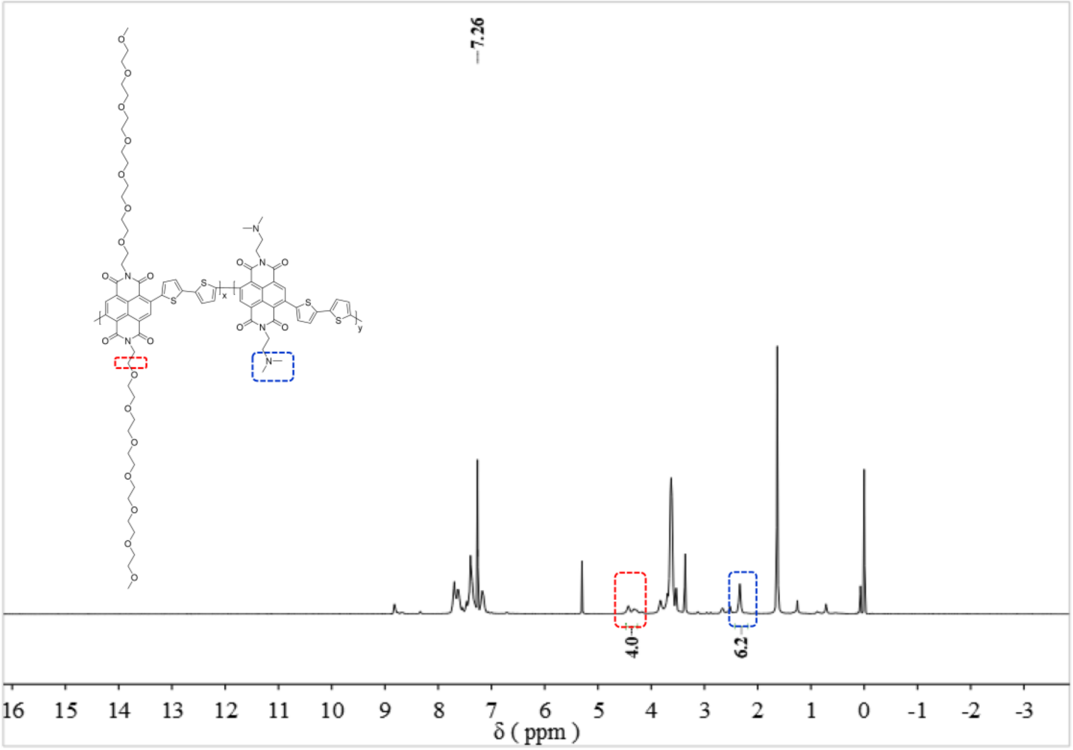

Supplementary Fig. 34 ^{1}H NMR spectrum of 2T-NDI-N-gly in $CDCl_3$. According to the hydrogen ratio in the blue and red boxes, the x: y of the polymer is 6:3.1.

Synthesis of BOH polymer.[14, 15] 2T-NDI-N-gly (20 mg), excess 4-(bromomethyl)phenylboronic acid (200 mg, 1 mmol), and 1,8-bis(dimethylamino)naphthalene (40 mg, 0.2 mmol) was suspended in DMF (10 mL) and stirred at 60 °C for 72 h. The resultant mixture was dissolved in hot methanol (45 ^{O}C) and the product was precipitated by adding Et_2O (50 mL). After cooling down to room temperature, the crude product was filtered directly and washed by THF (5 mL) for three times. The BOH polymer was obtained by vacuum drying (25 mg, 85% yield). The ^{1}H-NMR of BOH in D_2O only showed non-informative broad peaks.

Preparation of BOH NPs

1 mg of BOH polymers was dissolved in 2 mL of DMSO. Then the DMSO mixture was dispersed in 10 mL of ultrapure water and transferred into a dialysis tube (MWCO: 3500). The dialysis tube was placed in a large beaker with water (1 L) stirring for 1 day, and the water was changed every 6 hrs. Then the solution was condensed into 1 mL and stored in a 4 ^{o}C refrigerator.

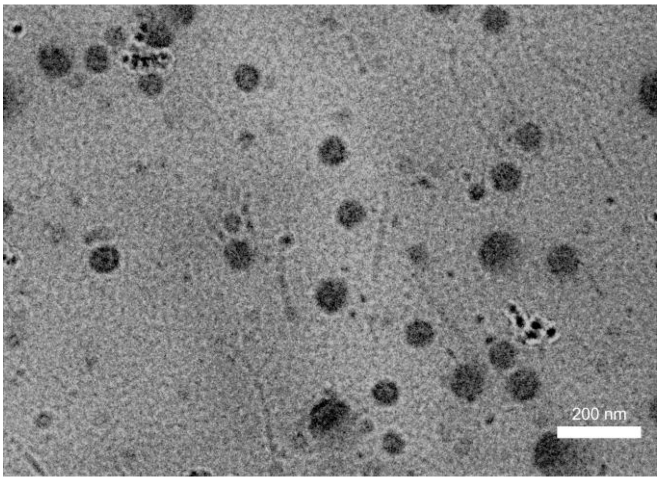

Supplementary Fig. 35 The TEM image of BOH NPs.

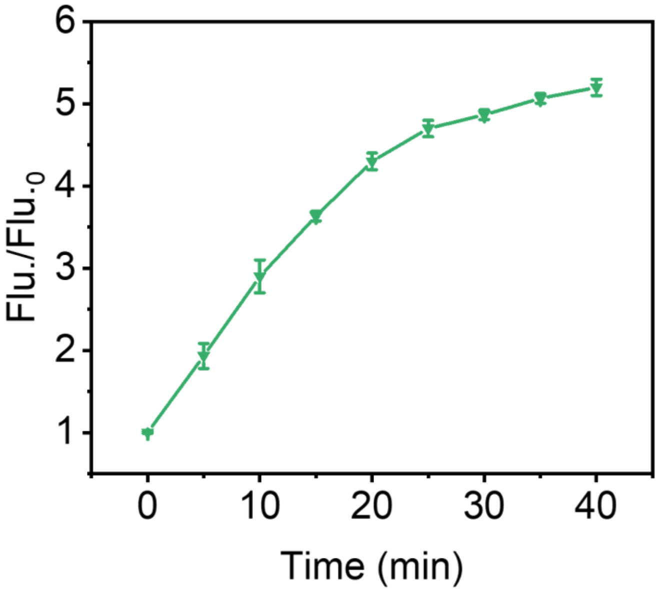

Supplementary Fig. 36 The kinetics of H_2O_2-induced NIR-II fluorescence change of the BOH NPs (5 μg mL^{-1}) showed a nearly complete probe activation within 40 min in the presence of H_2O_2 (10 μM in pH = 7.4 PBS buffer).

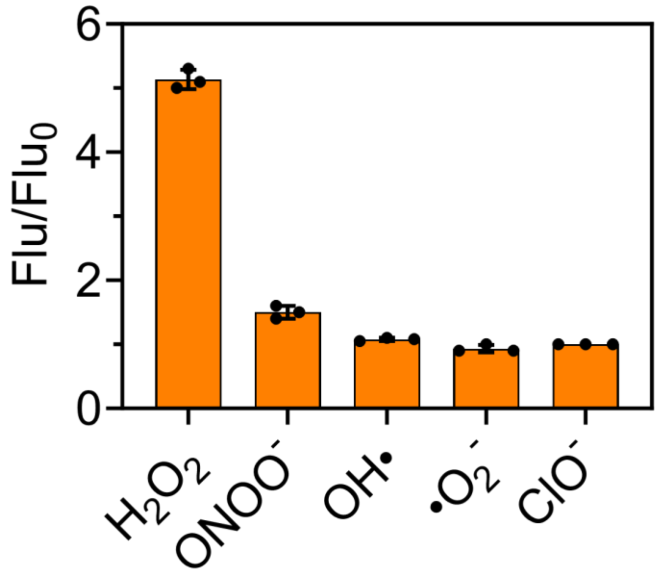

Supplementary Fig. 37 The selectivity study indicated that only H_2O_2 could dramatically cause the 940 nm emission to increase for the BOH NPs (5 μg mL^{-1}).

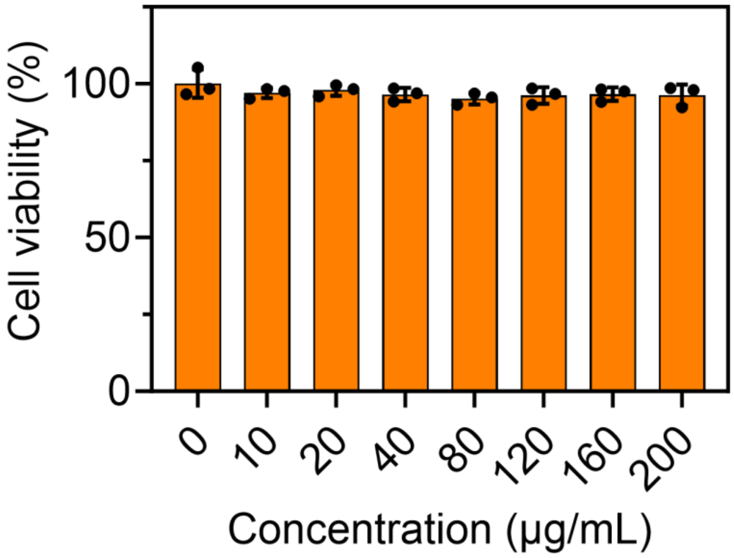

Supplementary Fig.38. *In vitro* viability of 4T1 cells treated with BOH NPs solutions at different concentrations for 24 h.

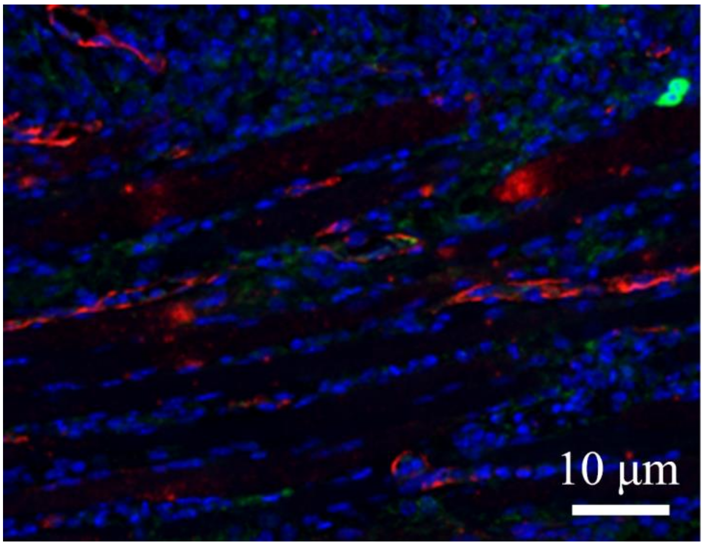

Supplementary Fig. 39. Immunofluorescence imaging of tumor slices. The tumor blood vessels (red) are stained with the anti-CD31 antibody, hypoxia-related protein HIF-α is stained with the anti-HIF-1α antibody (green).

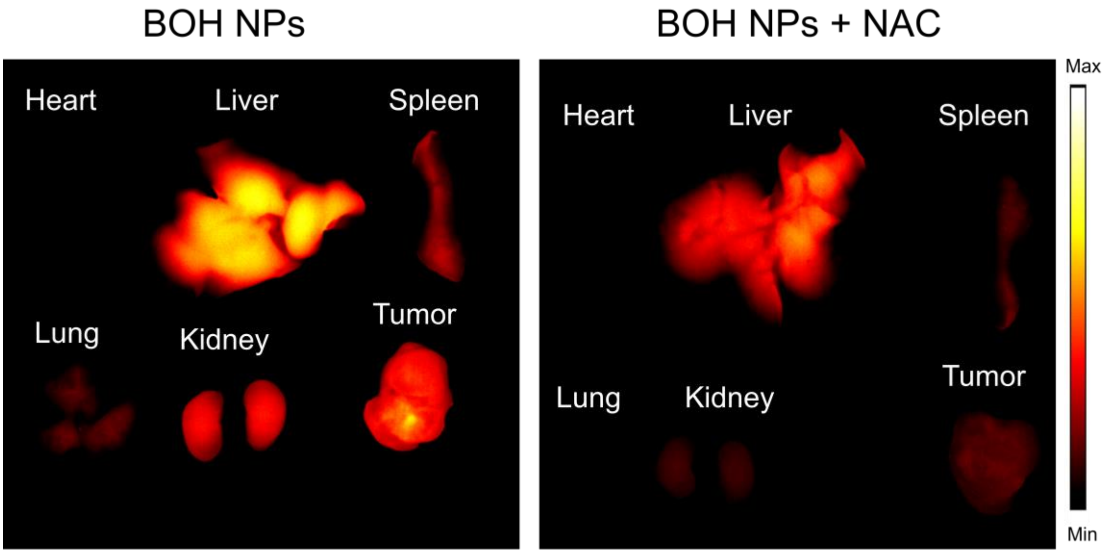

Supplementary Fig. 40 *Ex vivo* fluorescence images of major organs.

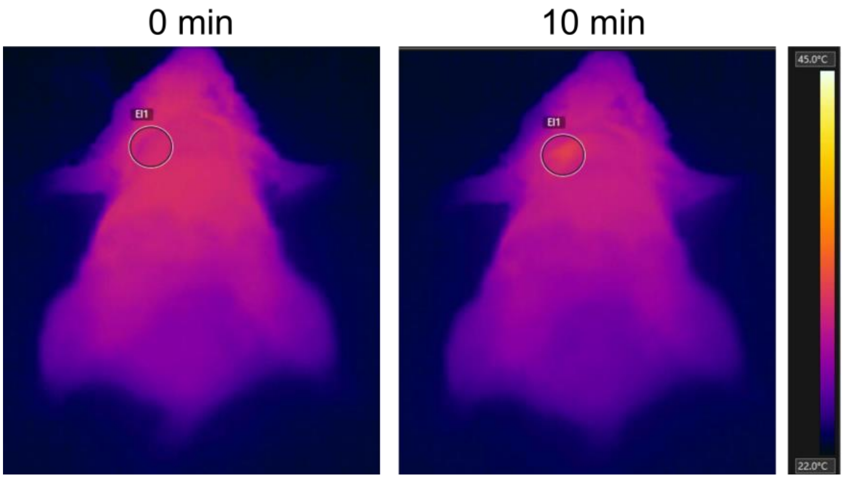

Supplementary Fig. 41 Thermal images of mice under continuous 808 nm laser irradiation (15 mW cm^{-2}, 10 min).

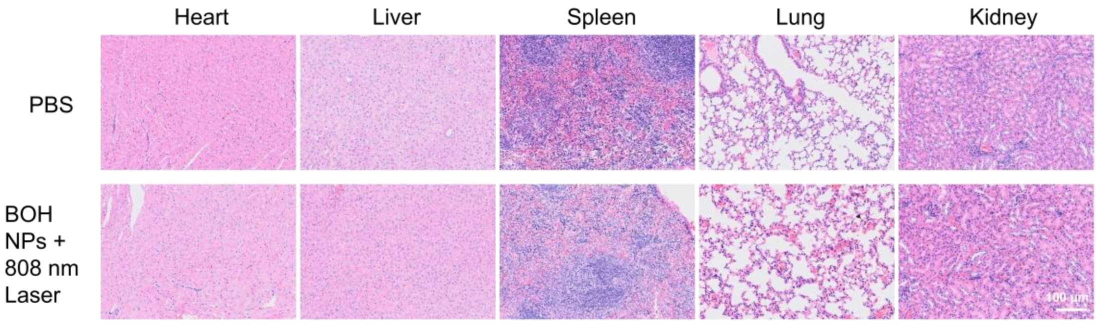

Supplementary Fig. 42 H&E staining of major organs.

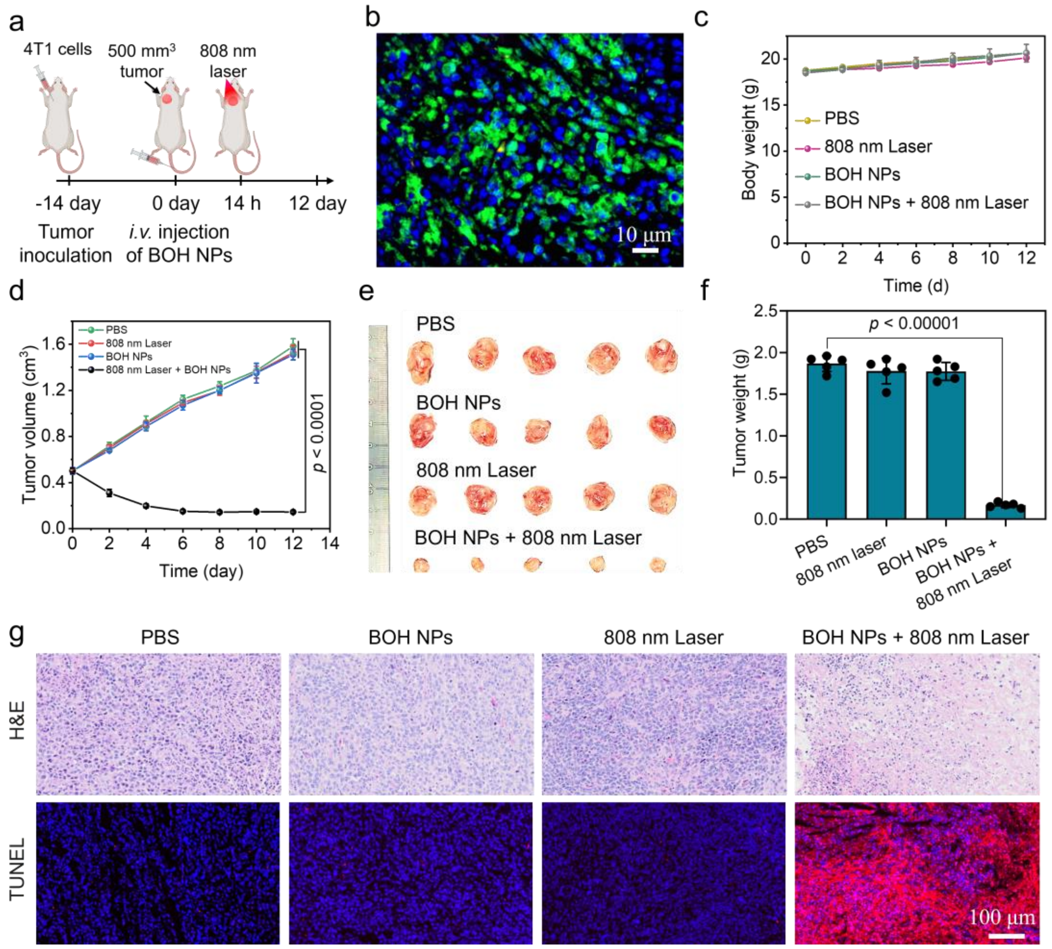

Supplementary Fig. 43 In vivo PDT evaluation of BOH NPs with large-size tumors (500 mm^3). (a) Schematic illustration of BOH NP-mediated PDT in a large volume of tumors (500 mm^3). (b) Immunofluorescence imaging of tumor slices. The tumor blood vessels (red) are stained with the anti-CD31 antibody, hypoxia-related protein HIF-α is stained with the anti-HIF-1α antibody (green). The (c) body weights and (d) tumor growth curves of the mice in vivo PDT study after intravenous injection of BOH NPs (1 mg mL^{-1}, 200 μL). The tumors of BOH NPs and BOH NPs + 808 nm groups were irradiated by an 808 nm laser with a power density of 15 mW cm^{-2} for 10 min. Mean ± SD, n = 5. ****p* < 0.0001. (e) Photos of tumors collected from the mice in different groups at the end of PDT. (f) The mean weight of tumors separated from mice after different treatments. Mean ± SD, n = 5. ****p* < 0.0001. (g) Representative H&E and TUNEL stained slices of tumors were collected from 4T1-tumor-bearing mice on the sixth day of the treatments in vivo PDT study.

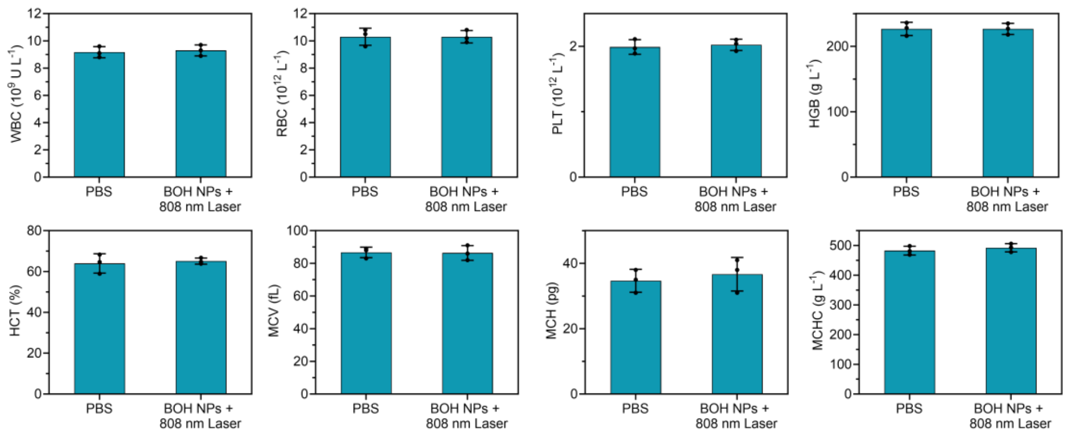

Supplementary Fig. 44 Haematological data analysis after the treatment sessions ended.

The haematological data include white blood cells (WBC), red blood cells (RBC), platelets (PLT), hemoglobin (HGB), hematocrit (HCT), mean corpuscular volume (MCV), mean corpuscular hemoglobin (MCH), and mean corpuscular hemoglobin concentration (MCHC). Compared with the control group, all the parameters in the BOH-NPs + 808 nm laser-treated groups did not show any significant differences, indicating that the therapy didn't cause obvious infection and inflammation in the treated mice.

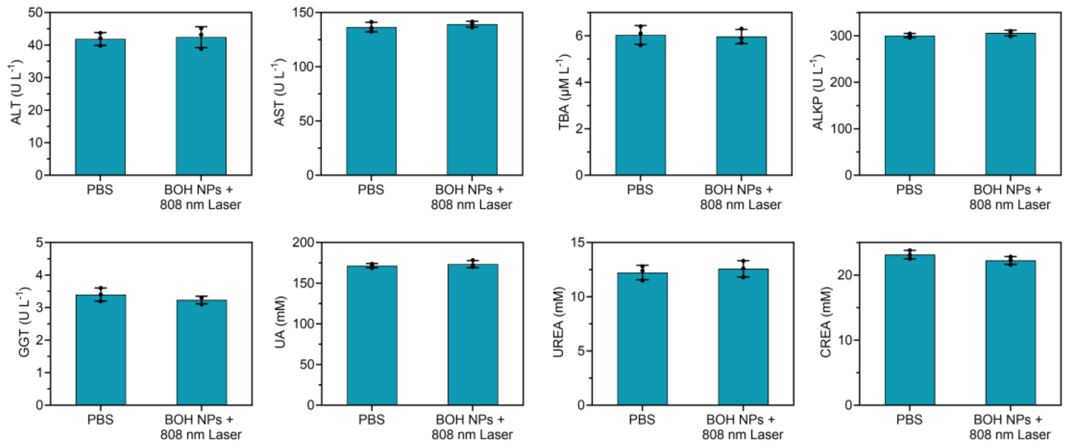

Supplementary Fig. 45 Blood biochemical assay after the treatment sessions ended.

Blood biochemical analysis, including Hepatic functional indexes (ALT, AST, TBA, ALKP and GGT) and renal function indexes (UA, UREA and CREA), were examined. No meaningful difference can be observed between the BOH-NPs + 808 nm laser-treated groups and the PBS control group, suggesting that the blood chemistry of mice is not affected by treatment. Furthermore, hepatic and renal functional indexes, such as ALT, AST and CREA, demonstrate that the therapy induces no significant adverse effects on basic liver and kidney in mice.

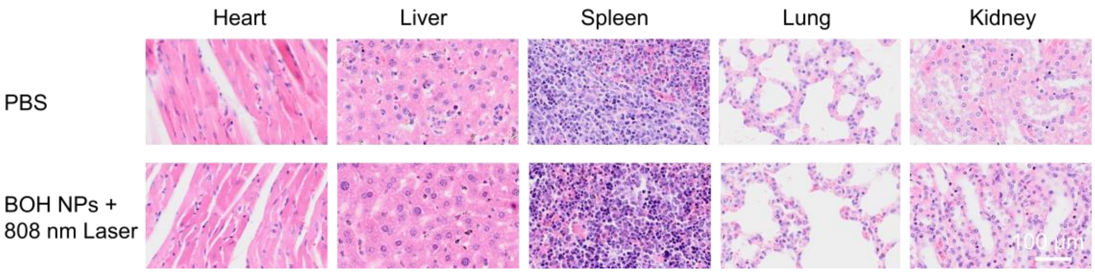

Supplementary Fig. 46 H&E data (haematoxylin and eosinstained images) obtained from the liver, spleen, kidney, heart and lung of the BOH-NPs-treated mice at 30 day post-injection. (Scale bar, 100 μm.)

H&E images of tissues (heart, liver, spleen, lung and kidney) harvested from PBS control mice and BOH-NPs + 808 nm laser-treated mice after the treatment sessions ended. The mice treated with buffered saline were used as blank controls (n = 3). No noticeable signal of organ damage can be observed after treatment from the two groups, suggesting no apparent histological abnormalities or lesions after the treatment.

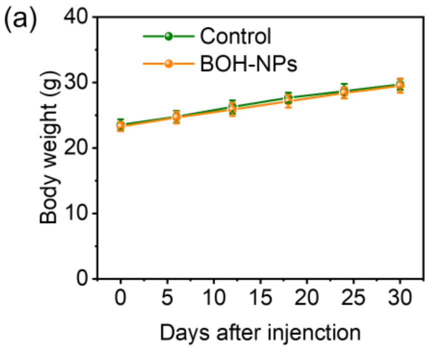

(b)

Behaviours and appearance	Eating			Drinking			Hair Colour			Glossiness		
	1 day	7 day	30 day	1 day	7 day	30 day	1 day	7 day	30 day	1 day	7 day	30 day
Control	No any significant differences			No any significant differences			No any significant differences			No any significant differences		
BOH-NPs												

Supplementary Fig. 47 (a) Body weight curve and (b) behaviours and appearance of BOH-NPs (100 mg kg^{-1}) treated mice. Body weight changes of healthy mice with and

without intravenous injections of BOH-NPs (n = 3 mice in each group) over 30 days. Data were presented as mean ± s.d.

To assess the influence of BOH-NPs on the development and growth of mice, body weight was continuously recorded. Each mouse was injected with 100 μL of buffered saline (control) or buffered probe BOH-NPs (dosage: 100 mg kg-1) by tail vein. Following these injections, the mice were weighed at different time points from 0 to 30 days. As shown in Supplementary Fig. 46, the mice injected with BOH-NPs showed no body weight loss compared with control mice without any treatment over the 30 days. Additionally, the mice did not show any significant differences in behaviours and appearance, including eating, drinking, hair colour and glossiness, between the BOH-NPs-treated mice and the control groups. These results demonstrated that BOH-NPs didn't cause overall side effects in the mice.

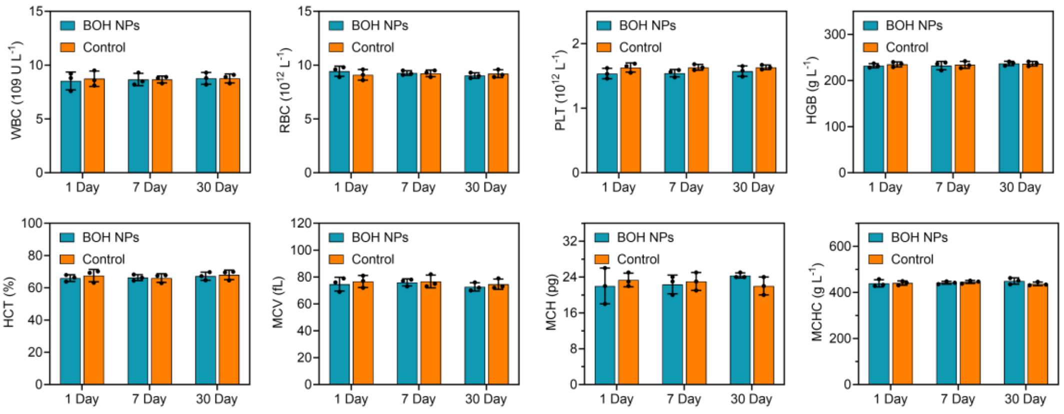

Supplementary Fig. 48 Haematological data analysis of the mice intravenously injected with the BOH-NPs at 1, 7 and 30 days post-injection.

The data include white blood cells (WBC), red blood cells (RBC), platelets (PLT), hemoglobin (HGB), hematocrit (HCT), mean corpuscular volume (MCV), mean corpuscular hemoglobin (MCH), and mean corpuscular hemoglobin concentration (MCHC). Compared with the control group, all the parameters in the BOH-NPs-treated groups at all-time points did not show any significant differences, indicating that the BOH-NPs didn't cause obvious infection and inflammation in the treated mice.

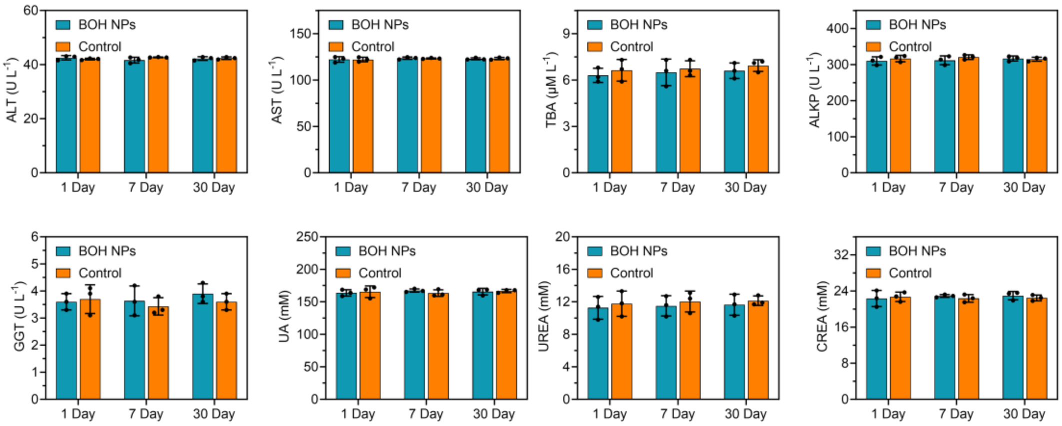

Supplementary Fig. 49 Blood biochemical assay of BOH-NPs.

Hepatic functional indexes (alanine transaminase (ALT), aspartate transaminase (AST), total bile acid (TBA), alkaline phosphatase (ALKP) and gamma-glutamyl transferase (GGT)) and renal function indexes (uric acid (UA), urea nitrogen (UREA) and creatinine (CREA)) of the mice treated with and without BOH-NPs. Blood biochemical analysis, including Hepatic functional indexes (ALT, AST, TBA, ALKP and GGT) and renal function indexes (UA, UREA and CREA), were examined. No meaningful difference can be observed between the BOH-NPs-treated groups at all time points with the control group, suggesting that the blood chemistry of mice is not affected by BOH-NPs treatment. Furthermore, hepatic and renal functional indexes, such as ALT, AST and CREA, demonstrate that the BOH-NPs induce no significant adverse effects on basic liver and kidney in mice.

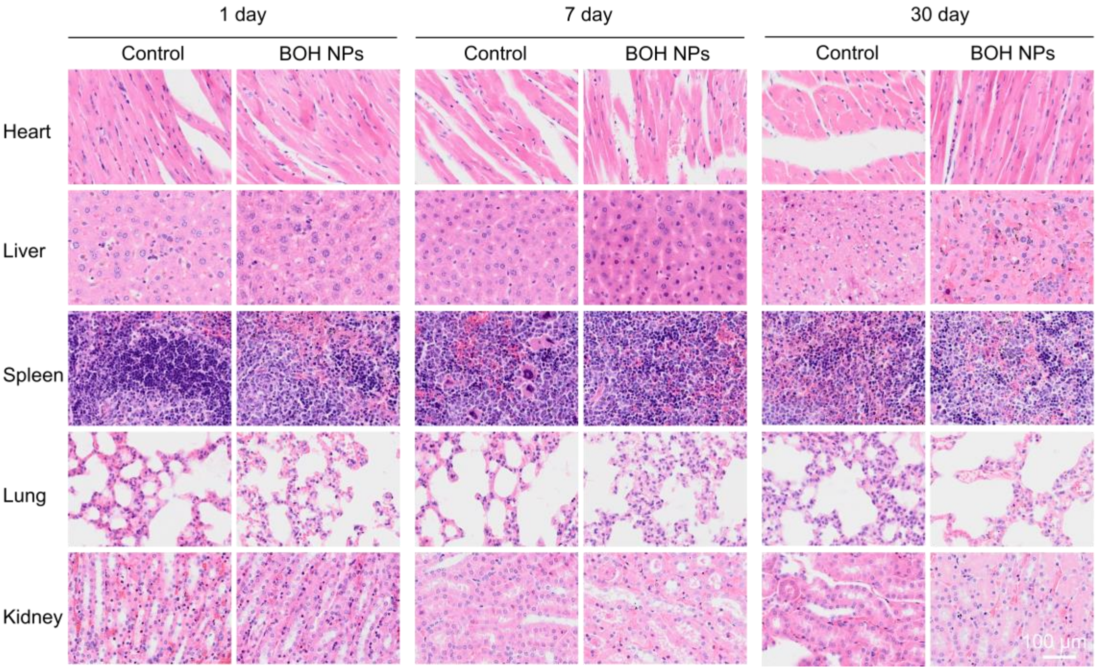

Supplementary Fig. 50 Histological data (haematoxylin and eosinstained images) obtained from the liver, spleen, kidney, heart and lung of the BOH-NPs-treated mice

at 1,7 and 30 day post-injection. (Scale bar, 100 μm.)

Haematoxylin and eosin-stained images of tissues (heart, liver, spleen, lung and kidney) harvested from control mice and 1, 7 and 30 day after intravenous injection of BOH-NPs (100 mg kg−1). The mice treated with buffered saline were used as blank controls (n = 3). No noticeable signal of organ damage can be observed during the whole treatment period from all the groups, suggesting no apparent histological abnormalities or lesions in the nanoprobe-treated groups for the test dose.

Supplementary methods

Materials

Unless otherwise noted, all reagents were purchased from Sigma Aldrich and used without additional purification. All other solvents were purchased from either Fisher Scientific or Aldrich and used as received.

Characterization

NMR spectra were recorded on a Bruker Ultra Shield Plus 400 MHz spectrometer (^{1}H: 400 MHz, ^{13}C: 101 MHz) and referenced to tetramethylsilane (TMS) as the internal standard. The absorption data and photoluminescence spectra were measured by a Shimadzu UV-3600 ultraviolet-visible-near-infrared spectrophotometer and an Edinburgh FLSP920 fluorescence spectrophotometer, respectively. All NIR-II fluorescence images were acquired on a home-built imaging set-up with a 319 × 256 pixels two-dimensional InGaAs array (NIRvana® 640 camera, Teledyne Princeton Instruments). Dynamic light scattering (DLS) studies were conducted using ALV/CSG-3 laser light scattering spectrometers at a scattering angle of 90°. Transmission electron microscopy (TEM) imaging was performed using an HT7700 transmission electron microscope operating at an acceleration voltage of 100 kV. The electrochemical cyclic voltammetry was recorded on an AUTOAB electrochemical analyzer with glassy carbon, Pt wire, and Ag/AgCl as working, counter and reference electrodes, respectively, in 0.10 M $(n\text{-}Bu)_4NPF_6$ acetonitrile at a scan rate of 50 mV/s. The GC-mass spectra were obtained from a Thermo Trace 1300GC+ ISQ MS with Msieve5A as a column. GPC measurements of the polymer in THF at flow rate of 1 mL min^{-1} under 40 ^{O}C. DFT calculations are performed at the ωB97XD*/6-31G(d) level by Gaussian 16, revision C.01.

Photocurrent Measurements

Electrochemical measurements were conducted using an AUTOAB electrochemical analyzer, employing a standard three-electrode configuration at room temperature. The working electrode was a glassy carbon electrode with a drop-casted polymer catalyst, possessing an effective area of 0.25 cm^2. A platinum electrode served as the counter electrode, while an Ag/AgCl electrode (3M KCl) was used as the reference electrode. The electrolyte solution consisted of 0.1 M KCl in aqueous medium. Before measurements, the system underwent a 10-minute N_2 purging to eliminate any traces of O_2. Photocurrents of the films were recorded under the illumination of an 808 nm laser, employing a 10-second on/off cycle.[16]

Mass spectra of the products in H_2O containing $H_2{}^{18}O$.

The coumarin (0.2 mg/mL) was added in NTOalk NPs (0.1 mg/mL) or NTgly NPs (0.1 mg/mL) solution in H_2O (0.5 mL) containing $H_2{}^{18}O$ (0.5 mL), respectively. Then the NTOalk NPs and NTgly NPs solutions were illuminated with an 808 nm laser for 20 min. Then the solution was used for mass spectra analysis.[17, 18]

Quartz Crystal Microbalance with Dissipation Monitoring (QCM-D)[19]

QCM-D measurements (QSense Analyzer) were performed with SiO_2-coated crystal sensors. The QCM-D response of bare sensors was monitored in water. Fluid injection into the chamber causes large changes in the QCM-D signals, which must be excluded from the mass uptake calculations. NTalk and NTgly were dissolved in chloroform at 10 mg/mL concentrations and spin-coated onto previously measured clean crystal sensors. As done with bare sensors, polymer-coated sensors were monitored in the water or without water. We compare the absolute difference among several overtones between the bare and the coated sensors using the “Stitch data” function of the Q-Soft software. Mass uptake was calculated using the Sauerbrey equation, which relates the changes in mass (Δm) to the frequency differences (Δf), using one overtone (n) as shown in the equation.

$$\Delta_m = \frac{-17.7}{n} \Delta f_n$$

Contact angle measurements

Contact angle measurements (Kruss DSA 20) were taken on thin polymer films spin-coated on polyethylene naphthalate substrates.

Detection of ROS production with ESR spectroscopy

Electron spin resonance (ESR) spectroscopy was employed to confirm the generation of $\bullet O_2^-$ and •OH. DMPO was used as a spin-trap agent for $\bullet O_2^-$ and •OH. ESR spectroscopy was employed to detect the ESR signals. Three PS NPs were 5 μg mL^{-1} under 808 nm irradiation at 15 mW cm^{-2} for 5 min under normoxia (21% O_2) or hypoxic (1% O_2) conditions.

Cell viability assay

The MTT assay was used to determine the in vitro cytotoxicity of PS in 4T1 cells at a density of 5 × 10^4 cells per well. The cells were inculcated in a medium containing different doses of PS for 24 h. After that, 10 μL of MTT (0.5 mg mL^{-1}) solution was added to each well. After 3 h incubation at 37 °C, the supernatant was removed, and 200 μL of dimethyl sulfoxide (DMSO) was added. A PowerWave XS/XS2 microplate spectrophotometer was used to record the absorbance at 490 nm. The cellular viability relative to the control group (PBS-treated cells) was calculated as A_{sample} /$A_{control}$, in which A_{sample} and $A_{control}$, respecti vely, represent the average absorption of groups containing PS and control cells.

PI and AM Staining

4T1 cells were cultured in laser confocal dishes at a density of 5 × 10^4 cells per well. Following 24 hours of incubation, BOH NPs (5 μg mL^{-1}) were introduced. After a 16-hour incubation period, the 4T1 cells within the confocal dish underwent 5-minute irradiation using an 808 nm laser at a power density of 15 mW cm^{-2}. The confocal dish was subsequently returned to the incubator for an additional 30 minutes. Following this, the 4T1 cells were rinsed thrice with PBS. Subsequently, PI (5 μg mL^{-1}) and AM (5 μg mL^{-1}) were applied for staining and allowed to incubate at 37 ℃ for 30 minutes. Following another round of triple PBS washing, an inverted fluorescence microscope was utilized to capture images.

A hypoxic environment in biological tests

We employed an AnaeroPack™-Anaero Anaerobic Gas Generator within a sealed transparent chamber. Cells were precultured in this controlled environment for 12 hours at 37 °C before initiating ROS detection. The ROS-ID™ hypoxia/oxidative stress detection kit was utilized in accordance with the product manual to signify the intracellular hypoxic condition. During the experiment, the co-incubation of BOH-NPs and the ROS detection probe with cells occurred within the hypoxic setting, with an oxygen concentration of approximately 1%. All other procedural steps remained consistent with those performed in a normoxic environment.

In vivo imaging

All tumor bearing nude mice were purchased from Jiangsu KeyGEN BioTECH Corp., Ltd. and used according to the guideline of the Laboratory Animal Center of Jiangsu KeyGEN BioTECH Corp., Ltd. Female BALB/c mice bearing 4T1 tumors with a tumor volume of 100 mm^3 were randomly divided into 2 groups (n = 3). Two groups of mice were intravenously injected with PBS (i.p., 25 μL) or NAC (i.p., 10 mg/kg in 25 μL of PBS), respectively. Six hours after the injection, all mice were intravenously injected with BOH NPs (1 mg mL^{-1}, 100 μL). Then, the fluorescence was recorded at different time points with an NIR-II fluorescence imaging system.

***Ex Vivo* Histological Staining.**

The hematoxylin-eosin (H&E) and the terminal deoxynucleotidyl transferase dUTP nick end labeling (TUNEL) stained slices of tumors were collected from 4T1-tumor-bearing mice on the sixth day of the treatments *in vivo* PDT study. The mice were sacrificed for tumor collection. The excised tumors were immersed in OTC and transferred for cryosection to obtain 10 μm thick slices for histological analysis. The H&E and the TUNEL staining assay were performed according to standard protocol. At the end of the treatment, the mice were sacrificed to collect organs for H&E staining. The observation was performed under a fluorescent microscope (Olympus inverted microscope IX-71).

In vivo therapeutic studies of large-size tumor

All tumor bearing nude mice were purchased from Jiangsu KeyGEN BioTECH Corp., Ltd. and used according to the guideline of the Laboratory Animal Center of Jiangsu KeyGEN BioTECH Corp., Ltd. Female BALB/c mice bearing 4T1 tumors with a tumor volume of 500 mm^3 were randomly divided into 4 groups (n = 5), including (i) PBS (ii) BOH NPs group, the 4T1 tumor-bearing mice were only intravenously injected with BOH NPs (1 mg mL^{-1}, 200 μL) without irradiation of 808 nm laser; (iii) 808 nm laser group, the mice was only irradiated under 808 nm laser with ultralow-power density (15 mW cm^{-2}) for 10 min at postinjection 12 h; (iv) BOH NPs + 808 nm laser group, the 4T1 tumor-bearing mice were intravenously injected with BOH NPs (1 mg mL^{-1}, 200 μL), then 808 nm laser irradiation with ultralow-power density (15 mW cm^{-2}) and short time (10 min) was performed at postinjection 12 h. During 14 days, mice were observed for changes in tumor growth and body weight. Tumor advancement was tracked by measuring individual tumor sizes using a digital vernier caliper. The formula used to calculate the individual tumor volume (V) was as follows: $V = (a \times b^2) / 2$. In this formula, 'a' represented the longer dimension of the tumor tissue, while 'b' represented the shorter dimension perpendicular to the length.

Investigation of the dark toxicity of BOH-NPs

All mice were purchased from Jiangsu KeyGEN BioTECH Corp., Ltd. and used according to the guideline of the Laboratory Animal Center of Jiangsu KeyGEN BioTECH Corp., Ltd. We used healthy C57Bl/6 mice (7 weeks old) to investigate the change in mice weight, hematology indicators, blood biochemical analysis, hepatic and renal functional indexes and haematoxylin and eosin staining analysis post-injection. Firstly, randomly selected 6 mice were randomly divided into 2 groups (n = 3 per group) and subjected to variable conditions, including (1) a control group without any treatment, (2) BOH-NPs (dose ~ 100 mg/kg) intravenously injected into the mice. Their body weight was monitored post-injection to assess healthy condition changes.

The 36 mice were randomly divided into 2 groups (n = 18 per group) and subjected to variable conditions, including (1) a control group without any treatment and (2) BOH-NPs (dose ~100 mg/kg) intravenously injected into the mice. The

haematological, blood biochemical and histological analyses were performed at time points of 1, 7 and 30 days post-injection, respectively. The mice were then sacrificed. The heart, lung, liver, spleen and kidney were embedded in paraffin, sectioned, and stained with hematoxilin and eosin (H&E), respectively. The tissues were subsequently processed for histopathological examination under a light microscope.

Supplementary References

1. Wan Q, *et al.* Molecular Engineering to Boost AIE-Active Free Radical Photogenerators and Enable High-Performance Photodynamic Therapy under Hypoxia. *Adv. Funct. Mater.* **30**, 2002057-2002069 (2020).

2. Laustriat G. Molecular mechanisms of photosensitization. *Biochimie.* **68**, 771-778 (1986).

3. Zhao X, *et al.* Molecular engineering to accelerate cancer cell discrimination and boost AIE-active type I photosensitizer for photodynamic therapy under hypoxia. *Chem. Eng. J.* **410**, 128133 (2021).

4. Lin Z, Zhang L, Tu S, Wang W, Ling Q. Highly thermally stable all-polymer solar cells enabled by photo-crosslinkable bromine-functionalized polymer donors. *Solar Energy* **201**, 489-498 (2020).

5. Sharman WM, Allen CM, van Lier JE. Photodynamic therapeutics: basic principles and clinical applications. *Drug Discov. Today* **4**, 507-517 (1999).

6. Teng KX, Niu LY, Xie N, Yang QZ. Supramolecular photodynamic agents for simultaneous oxidation of NADH and generation of superoxide radical. *Nat. Commun.* **13**, 6179 (2022).

7. Chen W, *et al.* Integration of TADF Photosensitizer as "Electron Pump" and BSA as "Electron Reservoir" for Boosting Type I Photodynamic Therapy. *J. Am. Chem. Soc.* **145**, 8130-8140 (2023).

8. Zhou B, Hu Z, Jiang Y, Zhong C, Sun Z, Sun H. Theoretical exploitation of acceptors based on benzobis(thiadiazole) and derivatives for organic NIR-II fluorophores. *Phys. Chem. Chem. Phys.* **20**, 19759-19767 (2018).

9. Takimiya K, Osaka I, Nakano M. π-Building Blocks for Organic Electronics: Revaluation of “Inductive” and “Resonance” Effects of π-Electron Deficient Units. *Chem. Mater.* **26**, 587-593 (2013).

10. Guo X, *et al.* Dialkoxybithiazole: a new building block for head-to-head polymer semiconductors. *J. Am. Chem. Soc.* **135**, 1986-1996 (2013).

11. Giovannitti A, *et al.* The Role of the Side Chain on the Performance of N-type Conjugated Polymers in Aqueous Electrolytes. *Chem. Mater.* **30**, 2945-2953 (2018).

12. Quek G, Vazquez RJ, McCuskey SR, Kundukad B, Bazan GC. Enabling Electron Injection for Microbial Electrosynthesis with n-Type Conjugated Polyelectrolytes. *Adv. Mater.* **34**, e2203480 (2022).

13. Moia D, *et al.* Design and evaluation of conjugated polymers with polar side chains as electrode materials for electrochemical energy storage in aqueous electrolytes. *Energy Environ. Sci.* **12**, 1349-1357 (2019).

14. Wu X, *et al.* Induced helical chirality of perylenebisimide aggregates allows for enantiopurity determination and differentiation of alpha-hydroxy carboxylates by using circular dichroism. *Chem* **20**, 11793-11799 (2014).

15. Chen XX, *et al.* Multicomponent covalent dye assembly for tight binding and sensitive sensing of L-DOPA. *Chem. Commun.* **51**, 13630-13633 (2015).

16. Wang S-H, *et al.* Solution-Processable Naphthalene Diimide-Based Conjugated Polymers as Organocatalysts for Photocatalytic CO2 Reaction with Extremely Stable Catalytic Activity for Over 330 Hours. *Chem. Mater.* **34**, 4955-4963 (2022).

17. Liao L, *et al.* Efficient solar water-splitting using a nanocrystalline CoO photocatalyst. *Nat. Nanotechnol.* **9**, 69-73 (2014).

18. Czili H, Horváth A. Applicability of coumarin for detecting and measuring hydroxyl radicals generated by photoexcitation of TiO2 nanoparticles. *Applied Catalysis, B: Environmental* **81**, 295-302 (2008).

19. Kosco J, *et al.* Oligoethylene Glycol Side Chains Increase Charge Generation in Organic Semiconductor Nanoparticles for Enhanced Photocatalytic Hydrogen Evolution. *Adv. Mater.* **34**, e2105007 (2022).
