# Supplementary material for: Oxygen-independent organic photosensitizer with ultralow-power NIR photoexcitation for tumor-specific photodynamic therapy
Source: Nat Commun. 2024 Mar 21;15:2530. doi: 10.1038/s41467-024-46768-w (PMC10957938; doi:10.1038/s41467-024-46768-w)
Supplement: Supplementary file 2 — Reporting Summary [file 41467_2024_46768_MOESM2_ESM.pdf]

nature portfolio

Corresponding author(s): Quli Fan, Bin Liu

Last updated by author(s): Feb 12th, 2024

Reporting Summary

Nature Portfolio wishes to improve the reproducibility of the work that we publish. This form provides structure for consistency and transparency in reporting. For further information on Nature Portfolio policies, see our Editorial Policies and the Editorial Policy Checklist.

Statistics

For all statistical analyses, confirm that the following items are present in the figure legend, table legend, main text, or Methods section.

n/a	Confirmed	
☐	☒	The exact sample size (n) for each experimental group/condition, given as a discrete number and unit of measurement
☐	☒	A statement on whether measurements were taken from distinct samples or whether the same sample was measured repeatedly
☐	☒	The statistical test(s) used AND whether they are one- or two-sided<br>*Only common tests should be described solely by name; describe more complex techniques in the Methods section.*
☐	☒	A description of all covariates tested
☒	☐	A description of any assumptions or corrections, such as tests of normality and adjustment for multiple comparisons
☐	☒	A full description of the statistical parameters including central tendency (e.g. means) or other basic estimates (e.g. regression coefficient) AND variation (e.g. standard deviation) or associated estimates of uncertainty (e.g. confidence intervals)
☐	☒	For null hypothesis testing, the test statistic (e.g. F, t, r) with confidence intervals, effect sizes, degrees of freedom and P value noted<br>*Give P values as exact values whenever suitable.*
☒	☐	For Bayesian analysis, information on the choice of priors and Markov chain Monte Carlo settings
☒	☐	For hierarchical and complex designs, identification of the appropriate level for tests and full reporting of outcomes
☒	☐	Estimates of effect sizes (e.g. Cohen's d, Pearson's r), indicating how they were calculated

Our web collection on statistics for biologists contains articles on many of the points above.

Software and code

Policy information about availability of computer code

Data collection	ZEISS LSM880 laser scanning confocal microscope for Confocal images. Living image software (version number, NIRvana® 640, Princeton Instruments) was used for the bioluminescence and fluorecence imaging.
Data analysis	Data mean ± standard deviation (SD) were calculated by Excel, Data Statistical analyses were performed by origin (version 2019) and GraphPad Prism 10.1.2.

For manuscripts utilizing custom algorithms or software that are central to the research but not yet described in published literature, software must be made available to editors and reviewers. We strongly encourage code deposition in a community repository (e.g. GitHub). See the Nature Portfolio guidelines for submitting code & software for further information.

Data

Policy information about availability of data

All manuscripts must include a data availability statement. This statement should provide the following information, where applicable:

- Accession codes, unique identifiers, or web links for publicly available datasets
- A description of any restrictions on data availability
- For clinical datasets or third party data, please ensure that the statement adheres to our policy

All the data supporting the findings of this study are available within the article and its Supplementary information files. The full image dataset is available from the corresponding author upon request. Source data are provided with this paper.

Research involving human participants, their data, or biological material

Policy information about studies with human participants or human data. See also policy information about sex, gender (identity/presentation), and sexual orientation and race, ethnicity and racism.

Reporting on sex and gender	No applicable
Reporting on race, ethnicity, or other socially relevant groupings	No applicable
Population characteristics	No applicable
Recruitment	No applicable
Ethics oversight	No applicable

Note that full information on the approval of the study protocol must also be provided in the manuscript.

Field-specific reporting

Please select the one below that is the best fit for your research. If you are not sure, read the appropriate sections before making your selection.

[x] Life sciences [ ] Behavioural & social sciences [ ] Ecological, evolutionary & environmental sciences

For a reference copy of the document with all sections, see nature.com/documents/nr-reporting-summary-flat.pdf

Life sciences study design

All studies must disclose on these points even when the disclosure is negative.

Sample size	The sample size of each experiment is indicated in the figure legends. Sample sizes were based on the authors' experience with in vitro and in vivo studies, as published in many studies. For animal models, experiments were designed to detect differences between imaging or treatment groups. Sample sizes may vary depending on animal availability. The sample size for cell-based assays was determined based on sample availability.
Data exclusions	No data were excluded.
Replication	All attempts at replication were successful. Experimental repeat numbers are also reported in Figure Legends
Randomization	All samples were randomly allocated into experimental groups.
Blinding	The investigators were blinded to group allocation during data collection and/or analysis. All experimental procedures and quantification of results, including injections, isolation of the tumors or organs, tissue histological analysis, were done by two independent researchers.

Reporting for specific materials, systems and methods

We require information from authors about some types of materials, experimental systems and methods used in many studies. Here, indicate whether each material, system or method listed is relevant to your study. If you are not sure if a list item applies to your research, read the appropriate section before selecting a response.

Materials & experimental systems

n/a	Involved in the study	
[ ]	[x]	Antibodies
[ ]	[x]	Eukaryotic cell lines
[x]	[ ]	Palaeontology and archaeology
[ ]	[x]	Animals and other organisms
[x]	[ ]	Clinical data
[x]	[ ]	Dual use research of concern
[x]	[ ]	Plants

Methods

n/a	Involved in the study	
[x]	[ ]	ChIP-seq
[x]	[ ]	Flow cytometry
[x]	[ ]	MRI-based neuroimaging

Antibodies

Antibodies used	Anti-CD31 antibody, Rabbit monoclonal (Catalog No. SAB5700639-100UL) was purchased from sigma
Validation	All antibodies used are commercially available and validated by the manufacturers, as indicated on the respective websites of

eachcommercial vendor, Please refer to the commercial website of each primary antibody for more details.

Eukaryotic cell lines

Policy information about cell lines and Sex and Gender in Research

Cell line source(s)	Breast cancer cell line (4T1 cells) was purchased from
Authentication	4T1 cells were not performed with authentication.
Mycoplasma contamination	No mycoplasma contamination was found.
Commonly misidentified lines (See ICLAC register)	No commonly misidentified cell lines were used.

Animals and other research organisms

Policy information about studies involving animals; ARRIVE guidelines recommended for reporting animal research, and Sex and Gender in Research

Laboratory animals	Female Balb/c mice (6-8 weeks old) and male C57BL/6 mice (6-8 weeks old) were purchased from Jiangsu KeyGEN BioTECH Corp., Ltd. All mice used in this study were maintained in a dedicated pathogen-free animal facility at 60 % of humidity and 25 degrees Celsius with 12/12 light schedule and free access to food and water.
Wild animals	The study did not involve wild animals.
Reporting on sex	All animal experiments were conducted on female mice,
Field-collected samples	The study did not involve samples collected from the field.
Ethics oversight	All tumor bearing nude mice were purchased from Jiangsu KeyGEN BioTECH Corp., Ltd. and used according to the guideline of the Laboratory Animal Center of Jiangsu KeyGEN BioTECH Corp., Ltd.

Note that full information on the approval of the study protocol must also be provided in the manuscript.

Plants

Seed stocks	No applicable
Novel plant genotypes	No applicable
Authentication	No applicable
